# Photochromic radical states in 3D covalent organic frameworks with zyg topology for enhanced photocatalysis

**DOI:** 10.1093/nsr/nwae177

**Published:** 2024-05-21

**Authors:** Tian-Tian Ma, Guo-Zhang Huang, Xiao-Han Wang, Yan Liang, Run-Han Li, Bin Wang, Su-Juan Yao, Jia-Peng Liao, Shun-Li Li, Yong Yan, Ya-Qian Lan

**Affiliations:** School of Chemistry, South China Normal University, Guangzhou 510006, China; Department of Chemistry, Guangdong Provincial Key Laboratory of Catalytic Chemistry, Southern University of Science and Technology, Shenzhen 518055, China; School of Chemistry, South China Normal University, Guangzhou 510006, China; School of Chemistry, South China Normal University, Guangzhou 510006, China; School of Chemistry, South China Normal University, Guangzhou 510006, China; School of Chemistry, South China Normal University, Guangzhou 510006, China; School of Chemistry, South China Normal University, Guangzhou 510006, China; School of Chemistry, South China Normal University, Guangzhou 510006, China; School of Chemistry, South China Normal University, Guangzhou 510006, China; School of Chemistry, South China Normal University, Guangzhou 510006, China; School of Chemistry, South China Normal University, Guangzhou 510006, China

**Keywords:** 3D covalent-organic frameworks, photochromic radical states, donor and acceptor, hydrogen peroxide, photocatalysis

## Abstract

Covalent-organic frameworks (COFs) with photoinduced donor-acceptor (D-A) radical pairs show enhanced photocatalytic activity in principle. However, achieving long-lived charge separation in COFs proves challenging due to the rapid charge recombination. Here, we develop a novel strategy by combining [6 + 4] nodes to construct **zyg**-type 3D COFs, first reported in COF chemistry. This structure type exhibits a fused Olympic-rings-like shape, which provides a platform for stabilizing the photoinduced D-A radical pairs. The **zyg**-type COFs containing catalytically active moieties such as triphenylamine and phenothiazine (PTZ) show superior photocatalytic production rates of hydrogen peroxide (H_2_O_2_). Significantly, the photochromic radical states of these COFs show up to 400% enhancement in photocatalytic activity compared to the parent states, achieving a remarkable H_2_O_2_ synthesis rate of 3324 μmol g^−1^ h^−1^, which makes the PTZ-COF one of the best crystalline porous photocatalysts in H_2_O_2_ production. This work will shed light on the synthesis of efficient 3D COF photocatalysts built on topologies that can facilitate photogenerating D-A radical pairs for enhanced photocatalysis.

## Teaser

Three-dimensional donor-acceptor covalent organic frameworks (COFs) with zyg topology featuring Olympic rings-shaped pores were constructed, showing metastable photoinduced charge-separated states which significantly enhanced the photocatalytic synthesis of hydrogen peroxide.

## INTRODUCTION

The photoinduced electron-transfer (PET) process plays an important role in the field of chemistry and biology, as exemplified by enzymatic catalysis [1], artificial photosystems [2] and solar energy conversion [3], among others [4,5]. Searching for new PET systems to produce long-lived charge separation states is crucial for the development of functional materials, especially photocatalysts. The charge separation in PET systems typically involves a redox process within the electron donor-acceptor (D-A) pairs, resulting in separated photoinduced radicals that possess stronger reducing or oxidizing ability and enhanced light absorption, in principle, compared with their neutral parent states [6–8]. The generation of photochromic radical states (PRSs) with lifetimes ranging from hours to days has been achieved in hybrid materials [9,10], supramolecular assemblies [11], metal-organic frameworks (MOFs) [12], hydrogen-bonded organic frameworks (HOFs) [13] and organic polymers [14], but is still absent in covalent organic frameworks (COFs). COFs have emerged as a new class of highly designable and tailorable crystalline porous organic skeletons with periodically ordered structures, showing great potential in heterogeneous catalysis on account of their high stability and porosity [15–18]. Thus, it is a crucial research direction to construct COF materials with PRSs in order to exploit the full extent of the photocatalytic performance.

**Figure 1.**
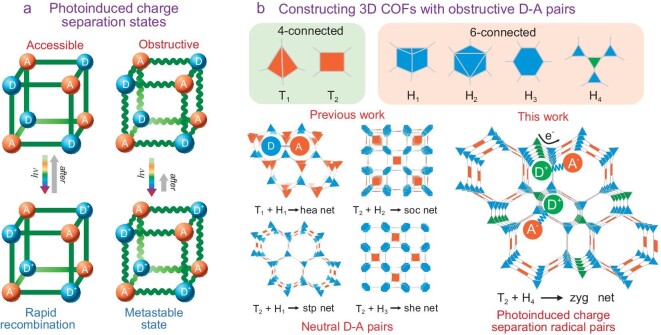
Schematic representation of (a) photoinduced charge separation of D-A pairs in 3D frameworks and (b) the 3D COFs constructed from 4-connected and 6-connected building blocks. Different from the reported [6 + 4] topologies, the **zyg** net can provide a platform for producing obstructive photoinduced charge separation radial pairs.

In the previously reported COFs, only the sub-second lifetime of the photoinduced charge separation (PCS) state can be reached [19–21]. The stabilization of long-lived charge separation states in COFs is confronted with two major challenges: one is to ensure the effective PET within the D-A pairs and the other is the inhibition of reversed charge recombination. In view of Marcus-Hush theory [22,23], regarding the former, distinct electron donating/accepting properties, suitable molecular orbit arrangement and close-in contact of the D-A pairs are required for effective electron jumping while the latter prefers a rather remote distance between the D-A pairs to prevent recombination. This means that building units with proper lengths are required for constructing such photochromic COFs. In view of the reticular chemistry [24,25], compared with the layer-by-layer stacking of 2D COF structures, the 3D interconnection of the building units in 3D COFs can afford more pathways for electron transfer and stabilize separated photoinduced charges through stronger electrostatic attraction interactions. In addition, 3D COFs with meso-porosity (with pore diameter larger than 2 nm) [26] can usually accommodate more accessible donor and acceptor sites with good catalytic activity [27]. Thus, 3D COF systems with suitable periodically ordered building blocks would be potential molecular platforms for the photogeneration of metastable charge separation radical states (Fig. 1a). Since the discovery of COFs by Yaghi in 2005 [15], most of the work has focused on the exploration of 2D structures and a small number of 3D COFs have been designed and synthesized [16,28,29]. In the 3D COFs database, the 4-connected building blocks with tetrahedral (*T_d_*) symmetry were mostly used to connect with other building units in [4 + 2], [4 + 3] and [4 + 4] modes to form topologies such as **dia, bor** and **pts** in the early studies [30–32]. In recent years, 3D COFs based on higher connectivity building units have been synthesized with new topologies featuring [6 + 2], [6 + 3], [6 + 4], [6 + 6], [8 + 2] or [8 + 4] nets [25]. Among these, 3D COF structures based on [6 + 4] net were constructed showing high surface area, high structural stability, an absence of structural interpenetration and decent catalytic properties. However, for [6 + 4]-connected COFs, only four topologies (**hea, soc, stp** and **she**) assembled by linking 6-connected building units with *T*_d_ and square nodes, have been reported, respectively (Fig. 1b) [33–36].

Triphenylamine (TPA) and phenothiazine (PTZ) are well-known electron-rich functional groups with distinct features, such as strong electron-donating characteristics, tunable redox properties and versatility of functionalization, meaning that they cater well to our purpose, acting as building units for 3D COFs with PCS states [37–39]. Herein, we have extended the triangular TPA and PTZ groups into (3,3)-type 6-connected building blocks, forming an irregular hexagon. Combining these designed hexagonal building units (hexa-CHO) with a planar quadrilateral building block (tetra-NH_2_), we have synthesized a family of (3,3,4)-connected 3D COFs with **zyg** topology, which has been previously observed in MOFs but is reported in COFs for the first time [40]. It is noteworthy that two types of 1D pores and an Olympic-rings-like shape are observed in the crystallographic axis *c* direction in the **zyg**-type 3D COFs. The rare multi-sized 1D pores have the potential to further increase the specific surface area, as well as to carry out some domain-limited catalytic reactions. This is the first 3D COF with a fused Olympic rings shape synthesized in COF structures. Most importantly, the metastable PCS radical states were obtained through light irradiation in the TPA- and PTZ-based COFs, confirmed by the photochromic phenomenon and electron paramagnetic resonance (EPR) characterization. To further investigate the more inherent impacts PCS brings to the COF materials, various photoelectrochemical characterizations were conducted revealing that light harvest ability and carrier transport capacity were both enhanced for the metastable radical states of the **zyg** COFs. In addition, TPA- and PTZ-based COFs often show high crystallinity, which can improve the photoelectron generation and transfer within the lattice [41]. Hydrogen peroxide (H_2_O_2_) is used as a green, clean and storable oxidant in a wide range of applications such as medical disinfection, paper bleaching and organic synthesis [42]. Herein, the TPA- and PTZ-based COFs were applied to the photocatalytic synthesis of H_2_O_2_, showing significant catalytic activity improvement for the metastable radical states (>200% enhancement for TPA COF and >400% for PTZ COF compared with the as-prepared samples) and the highest H_2_O_2_ photosynthesis rate reaches 3324 μmol g^−1^ h^−1^ for the activated PTZ-based COF, making it one of the best porous crystalline photocatalysts in H_2_O_2_ photosynthesis. This work has successfully constructed metastable charge separation radical states in COFs of **zyg** topology for the first time and enhanced the catalytic performance by several orders of magnitude, providing an effective strategy on catalytic activity enhancement for 3D COF materials.

## RESULTS AND DISCUSSION

### Synthesis and characterizations

**Figure 2.**
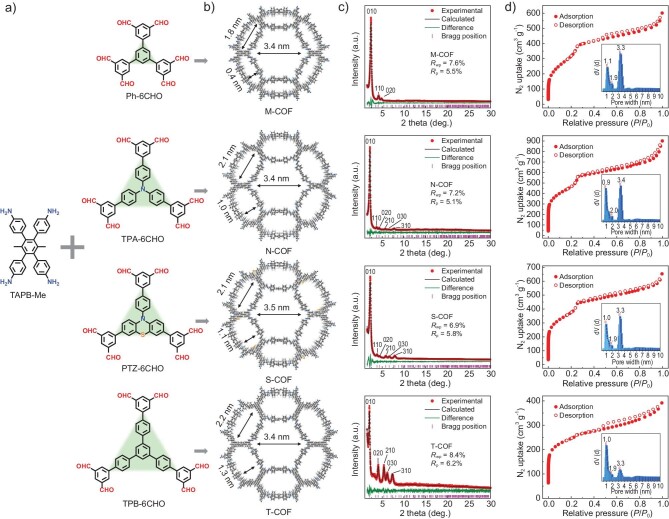
Structures and characterizations of **M-COF, N-COF, S-COF** and **T-COF**. (Columns b, c, d from top to bottom are in the order of **M-COF, N-COF, S-COF** and **T-COF**). (a) Synthesis of COFs using the hexa-aldehyde and tetra-amine precursors. (b) Extended structures of COFs viewed along the crystallographic *c* axis. (c) PXRD patterns and Pawley refinements of the unit cell parameters based on the crystal structure models. (d) N_2_ adsorption–desorption isotherms at 77 K and pore size distributions.

In order to construct new [6 + 4]-connected COFs, as shown in Fig. 2, hexa-aldehyde precursors containing functionalized central cores acting as strong electron donors (TPA-6CHO, PTZ-6CHO) and weak ones (Ph-6CHO, TPB-6CHO) were used. The crystalline products, named **M-COF, N-COF, S-COF** and **T-COF**, were obtained through the Schiff condensation reaction using the above aldehydes and dimethyl functionalized tetra-amine (TAPB-Me). These COF materials were synthesized in yields ranging from 60% to 70% via the solvothermal method, where the reactant monomers were heated at 120°C in a mixture containing *o*-dichlorobenzene, dichloromethane and trifluoroacetic acid for 3 days (see Supplementary Information for more details). The resulting four COFs were insoluble in common organic solvents such as dimethyl sulfoxide (DMSO), acetone and methanol (Figs S1 and S2, see Supplementary Information).

**Figure 3.**
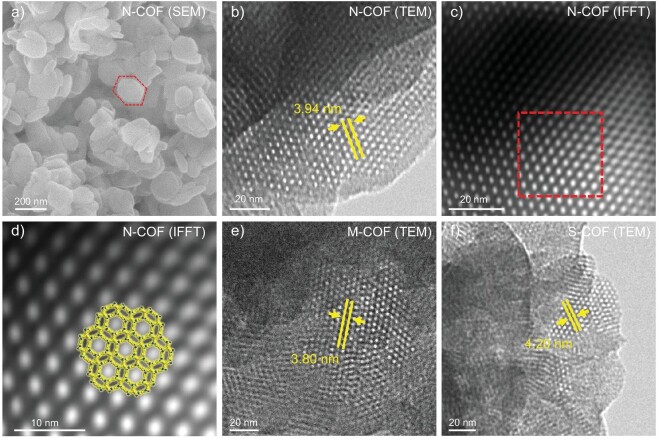
Electron microscopy images of the as-synthesized COFs. (a) SEM image of **N-COF**. A hexagonal crystal is marked with the red hexagon. (b) TEM image of **N-COF** and (c) its IFFT. The magnified region in the area marked with the red box in the IFFT image for **N-COF** (c) and overlay of the structure model along the [001] direction (d). TEM images of (e) **M-COF** and (f) **S-COF**.

The structures of these 3D COFs were unambiguously characterized using various analytical methods. Solid-state ^13^C cross-polarization magic angle spinning (CP/MAS) nuclear magnetic resonance (NMR) spectra (Figs S3–S6) verified the presence of the imine moiety showing peaks at ∼160 ppm. Considering that monomers with multiple reaction sites may give sub-stoichiometric COFs, in order to quantitatively determine the ligand ratios, these COFs were decomposed and dissolved using DCl/DMSO-*d*_6_ and analyzed by solution-state ^1^H NMR (Figs S7 and S8). The ratios of amino and aldehyde precursors in both **N-COF** and **S-COF** were close to 3:2. This stoichiometry is consistent with that observed in the crystal structure models. The Fourier transform infrared (FT-IR) spectra of **M-COF, N-COF, S-COF** and **T-COF** (Figs S9–S12) show the formation of imine bonds (1623 cm^−1^) and the disappearance of C–O vibration of the aldehyde group (1698 cm^−1^) and the N–H vibration of the amino group (3352 cm^−1^). Thermogravimetric analysis (TGA) (Fig. S13) was performed under N_2_ atmosphere to assess the thermal stability, and these 3D COFs were stable up to 450°C before being decomposed. Field emission scanning electron microscopy (SEM) images (Fig. 3a and Fig. S14) reveal that all these COFs show defined hexagonal nanocrystals.

### Crystalline structure and porosity

Measurements of powder X-ray diffraction (PXRD) combined with theoretical structural simulations were used to elucidate the crystal structures of the above COFs. The structural models for all these COFs were established based on **zyg** topology in the *P*6_3_/*mcm* space group. As shown in Fig. 2c, the experimental PXRDs for these four COFs exhibit the first strongest peaks at 2.40°, 2.14°, 2.20° and 2.04°, respectively, which are attributed to the [010] planes of the hexagonal unit cells. The calculated PXRD patterns of the geometrically optimized structures are in good agreement with the experimental results. Full profile Pawley refinements were performed to obtain the unit cell parameters (*a* = *b* = 42.39 Å, *c* = 9.15 Å, *R*_p_ = 5.5%, *R*_wp_ = 7.6% for **M-COF**; *a* = *b* = 47.65 Å, *c* = 9.65 Å, *R*_p_ = 5.1%, *R*_wp_ = 7.2% for **N-COF**; *a* = *b* = 46.87 Å, *c* = 9.26 Å, *R*_p_ = 5.8%, *R*_wp_ = 6.9% for **S-COF**; *a* = *b* = 50.08 Å, *c* = 8.91 Å, *R*_p_ = 6.2%, *R*_wp_ = 8.4% for **T-COF**), which led to satisfactorily low residual values and acceptable profile differences. Also, Rietveld refinements based on the **zyg**-type structural models for these COFs against the PXRD data revealed a good match between the calculated and experimental patterns (Figs S15–S22), confirming the model structures. According to the Reticular Chemistry Structure Resource (RCSR) [24], combining a potential co-planar 6-connected node with a square building block can also form the **rht**-network, which has been observed in a series of {Cu_2_(COO)_4_} paddlewheel-based MOFs [43–45]. Therefore, as an example, the crystal structure for **N-COF** was also modeled based on **rht** topology, where the three branched isophthalaldehydes of the hexa-CHO are on the same plane. However, the calculated PXRD pattern does not match with the experimental one (Fig. S23), which rules out the possibility for these COFs to adopt **rht** topology. In addition, the [6 + 4]-connected network with **stp** topology showing the same hexagonal symmetry as the **zyg**-net has been tested for the structural modeling of **N-COF**. The structure of **N-COF-stp** shows a significantly larger unit cell (space group: *P*_6_, *a* = 52.30 Å, *c* = 13.88 Å) than the one provided by the indexing of the experimental PXRD pattern, suggesting the network of **N-COF** adopts **zyg** topology rather than **stp** (Fig. S24). Furthermore, the crystallinity and periodic porous structures of these COFs were also evidenced by high-resolution transmission electron microscopy (TEM) (Fig. 3b–f and Figs S25–S27). The TEM images for **M-COF, N-COF** and **S-COF** all clearly reveal long-range structural ordering consisting of hexagonal honeycomb lattices viewed from the [001] direction. The periodic bright spots in these images associate with the larger empty pore channels of these COF structures. The corresponding inverse fast Fourier transform (IFFT) denoised images clearly show lattice fringes with distances of 3.80 nm for **M-COF**, 3.94 nm for **N-COF** and 4.20 nm for **S-COF**, matching well with the d-spacings of [100] lattice planes in these **zyg**-type frameworks. Micro electron diffraction (MicroED) experiments were also conducted on these COF nanocrystals in order to obtain the single crystal structures for these COFs. However, these COF samples showed barely any diffraction spots for single crystal structure solution from MicroED (Fig. S28).

**Figure 4.**
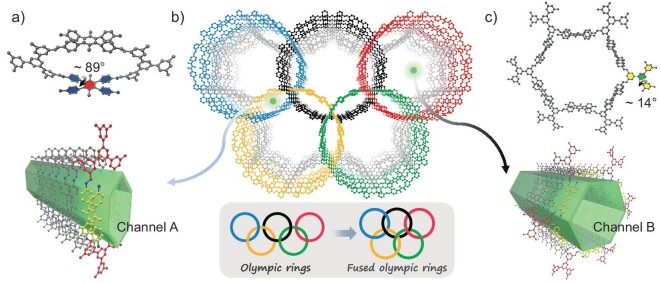
Structure of the fused-Olympic-rings-shaped framework and the two different channels in the **zyg**-type COFs exemplified by **M-COF**. (a) Single and multilayer aperture structures for the smaller hexagon (channel A). (b) Extended structure of **M-COF** viewed along the *c* axis and fused five-member rings derived from Olympic rings. (c) Single and multilayer aperture structures for the larger hexagon (channel B).

In the construction of the **zyg** net, the TAPB-Me unit acts as a square building block, in which the four branched phenyl rings are almost perpendicular to the central phenyl ring (Fig. 4a). The tetra-amine TAPB (1,2,4,5-tetrakis-(4-aminophenyl)benzene) without methyl groups was also tested to react with the same hexa-aldehydes, but no crystalline products were obtained, suggesting the important role of the two methyl groups in TAPB-Me for directing the formation of the **zyg**-type frameworks. In these **zyg**-type COFs, the three branched isophthalaldehyde rings of the hexa-CHO are not on the same plane, but with a small twist between the branched ring and the central phenyl ring (i.e. ∼14° for **M-COF**, Fig. 4c). The **zyg**-COFs can be seen in the unit cell *c* direction with two different sizes of pores, both of which can be simplified into hexagons (Channel A and B as shown in Fig. 4a and c, respectively). The irregular hexagon of Channel A is formed by two hexa-CHO units and two TAPB-Me moieties as the edges. Channel B is composed of six TAPB-Me units as edges connected with six isophthalaldehyde units as vertices to form a regular hexagon. Therefore, changing the size of the hexa-CHO building block affects the size of Channel A. The crystal structure of the expanded **M-COF** shows that the **zyg** network exhibits the shape of fused Olympic rings in the unit cell *c* axis (Fig. 4b). By varying the size of the hexa-CHO unit, the above four **zyg**-type COFs show different sizes of fused Olympic rings. To investigate the porosity of these 3D COFs, nitrogen adsorption analysis was performed at 77 K after activation of the materials under vacuum (Fig. 2d). The adsorption curves all exhibit typical type IV isotherms, showing both microporous and mesoporous properties. The surface areas for these COFs were calculated to be 1226 (**M-COF**), 1696 (**N-COF**), 1383 (**S-COF**) and 891 m^2^ g^−1^ (**T-COF**) according to the Brunauer-Emmett-Teller (BET) theory (Figs S29–S32). Based on non-local density functional theory (NLDFT), these COFs have corresponding pore size distribution peaks near 1.0 and 3.4 nm, which are essentially similar to the pore sizes expected from their crystal structures.

### Photophysical properties

**Figure 5.**
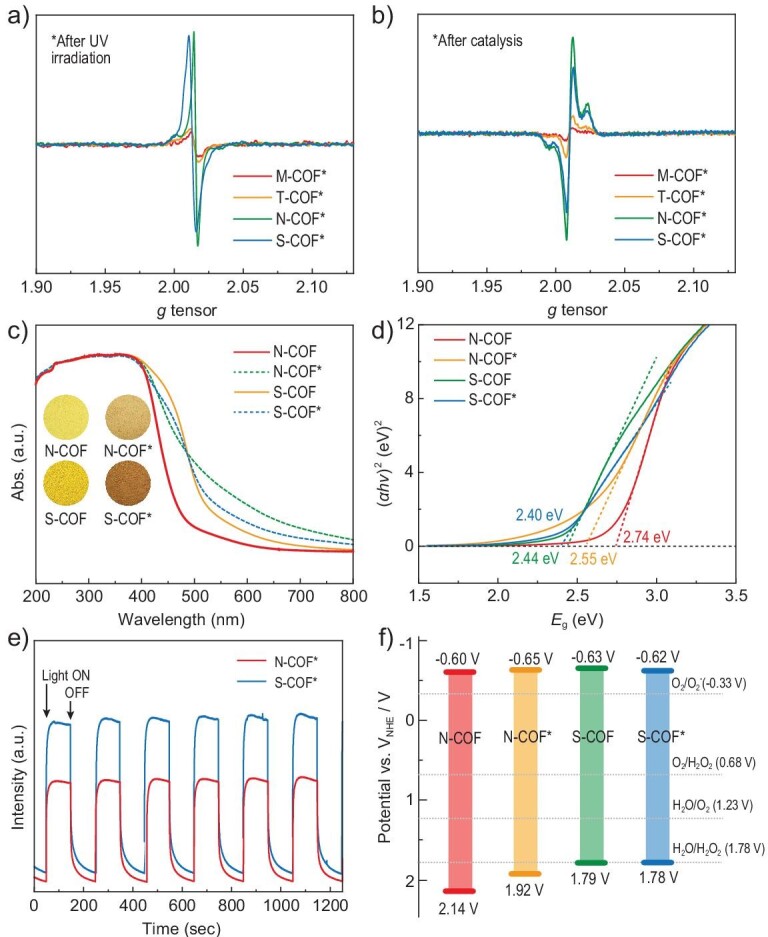
(a) EPR spectra for the irradiated samples of as-prepared **zyg**-type COFs in the solid state. (b) EPR spectra for the irradiated samples of as-prepared **zyg**-type COFs under the catalytic reaction condition. (c) UV-Vis DRS for **N-COF** and **S-COF** and their activated states (inset: photographs of the COF powders). (d) *T*_auc_ plots for band gap calculations. (e) Transient photocurrent response and (f) band-structure diagram for N-COF, S-COF, N-COF* and S-COF*. NHE stands for normal hydrogen electrode.

The structure feature of these COFs provides a geometric platform for stabilizing the charge separation states in a 3D lattice. Therefore, PCS in these COFs was investigated. First, the as-prepared COFs were irradiated by Xe lamp to induce the electron transfer within the COF. All the COF samples showed photochromism, and therefore solid-state EPR spectra were collected before and after light irradiation. When irradiating solid samples or samples dispersed in solution, only a subtle increase in the EPR signals around *g*-value of 2.0 was observed for **M-COF** and **T-COF** after light activation, indicating inefficient electron transfer or rapid charge recombination in them (Fig. 5a and b, and Figs S33 and S34). The EPR signals obviously emerged for the photo-activated samples of **N-COF** and **S-COF**, namely N-COF* and S-COF*, suggesting that the effective electron transfer process occurred and metastable PCS states formed inside **N-COF** and **S-COF** (Fig. 5a and b, and Figs S35 and S36). The electronic properties of **N-COF** and **S-COF**, as well as their activated samples, were subsequently investigated via various measurements. The yellow-colored samples of **N-COF** and **S-COF** showed slightly different photon capture ability, as revealed by their UV-Vis diffuse reflectance spectra (DRS). As seen in Fig. 5c, both **N-COF** and **S-COF** show broad absorption bands with maximum peaks at 380 nm and **S-COF** possesses better visible-light harvesting properties with a 50 nm wavelength red shifting. The change on UV-Vis DRS mainly results from the introduction of the phenothiazine group, increasing the conjugation property in the structure. The DRS also revealed that the metastable states N-COF* and S-COF* possess better visible light absorption ability, resulting from the radical feature of these activated states. By analyzing the *T*_auc_ plots of (*αhν*)^2^ vs. photon energy (Fig. 5d), the band gaps *E*_g_ were obtained as 2.74 eV and 2.44 eV for **N-COF** and **S-COF**, and 2.55 eV and 2.40 eV for N-COF* and S-COF*, respectively. For the purpose of verifying the energy band positions, Mott-Schottky (M-S) electrochemical measurements were also carried out. Based on the M-S results (Figs S37–S40), the highest occupied molecular orbital (HOMO) positions of **N-COF, S-COF** and their corresponding activated structures were calculated to be −0.60, −0.65, −0.63 and −0.62 eV, respectively. On the basis of the equation *E*_g_ = *E*_VB_ − *E*_CB_, the lowest unoccupied molecular orbital (LUMO) positions of N-COF, S-COF, N-COF* and S-COF* were calculated to be 2.14, 1.79, 1.92 and 1.78 eV, respectively. As shown in Fig. 5f, the band structures of **N-COF** and **S-COF** before and after activation were adequate for the synthesis of H_2_O_2_ from H_2_O (*E* (H_2_O_2_/H_2_O) = +1.78 V vs. NHE) and O_2_ (*E* (O_2_/H_2_O_2_) = +0.68 V vs. NHE), indicating that these COFs are suitable for the catalytic synthesis of H_2_O_2_.

It can be inferred above that the light activation of **N-COF** and **S-COF** samples increased the photon capture abilities and meanwhile narrowed the energy band gaps. To further understand the impact the PCS state brought to these two COFs, the transient photocurrent response was measured to evaluate the photogenerated carrier separation efficiency and the charge transport capacity. The transient-photocurrent-response intensity of **S-COF** was slightly larger than that of **N-COF** under visible light (*λ* > 420 nm). When illuminating **N-COF** and **S-COF** with an Xe lamp without any optical filter, the photocurrent showed intensity enhancement with illumination time increasing, meaning that the PCS states for these COFs benefit the photogenerated carrier separation efficiency and the charge transport capacity, and this trend is more significant for **S-COF** than **N-COF** (Fig. 5e, and Figs S41 and S42).

### Photocatalytic synthesis of H_2_O_2_

**Figure 6.**
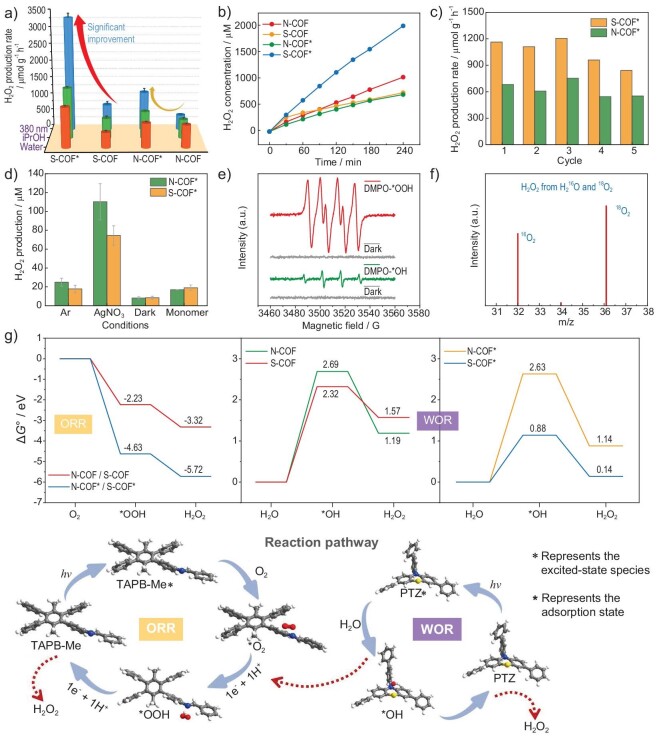
(a) Photocatalytic H_2_O_2_ production rates of N-COF, S-COF, N-COF* and S-COF* in pure water and 10% isopropanol aqueous solution. (b) Time-dependent photocatalytic activity of N-COF, S-COF, N-COF* and S-COF* for H_2_O_2_ production in pure water. (c) Cycling tests over N-COF* and S-COF* for the photocatalytic H_2_O_2_ production from O_2_ and H_2_O. (d) Single-variable control experimental tests of the photocatalytic H_2_O_2_ production performance for N-COF* and S-COF* under different conditions. (e) EPR spectra of the reaction solution under dark and visible light illumination for S-COF* in the presence of DMPO as the spin-trapping reagent. (f) ^18^O_2_ isotope experiment to identify the source of O in H_2_O_2_. (g) Free energy diagram of H_2_O_2_ photosynthesis through the ORR and WOR pathways for N-COF, S-COF, N-COF* and S-COF* and the key steps of the H_2_O_2_ photosynthesis mechanism.

Compared to the industrial anthraquinone oxidation method, the photocatalytic synthesis of H_2_O_2_ is safer and more environmentally friendly as it utilizes renewable solar energy and clean resources (H_2_O and O_2_) [42,46]. Currently, organic polymer-based photocatalysts such as graphitic carbon nitride (g-C_3_N_4_) have been widely applied in the photocatalytic production of H_2_O_2_ [47,48]. However, their further development has been hindered by the unsatisfactory catalytic activity due to the low crystallinity and poor photogenerated carrier separation. Since 2020, COFs have arisen as an ideal candidate for H_2_O_2_ photosynthesis because of their well-defined structures, excellent stability and desired semiconductor-like behavior [46,49]. Although the light absorption ability of these COFs is quite moderate, the fine photogenerated carrier separation efficiency and the charge transport capacity, as well as the suitable arrangement of the acceptor-donor pairs, make it possible for them to be used as highly efficient photocatalysts. Therefore, **N-COF** and **S-COF** containing photocatalytically active groups (TPA and PTZ) were tested for the synthesis of H_2_O_2_ (Figs S43–S48). First, the photocatalytic activity of the reported COFs was evaluated by photocatalytic oxygen reduction reaction (ORR) for H_2_O_2_ production under Xe lamp irradiation using isopropanol as a sacrificial reagent. The photosynthetic rates of H_2_O_2_ in 10% isopropanol aqueous solution are 560 and 596 μmol g^−1^ h^−1^ for **N-COF** and **S-COF**, respectively, while the values increased to 794 and 1474 μmol g^−1^ h^−1^ for N-COF* and S-COF* (Fig. 6a). The enhancement of photocatalytic performance might result from the better photon capture ability, photogenerated carrier separation efficiency and the charge transport capacity for the PCS radical samples. The much more dramatic increase in activity for the activated **S-COF** compared with **N-COF** can be attributed to the increase in conjugation property because of the phenothiazine group. Subsequently, H_2_O_2_ photocatalytic synthesis with an Xe lamp was also conducted in pure water, displaying H_2_O_2_ photosynthetic rates of 656, 450, 710 and 1160 μmol g^−1^ h^−1^ for N-COF, S-COF, N-COF* and S-COF*, respectively (Fig. 6a). There was a clear accumulation of the H_2_O_2_ yield with an increase in irradiation time, displaying a close linear relationship between H_2_O_2_ production and irradiation time for all the above COFs, suggesting that the high photosynthetic rates could remain even with a long irradiation time (Fig. 6b). This provides proof for the fact that the light-activated states were quite stable even in the catalytic reaction condition. Furthermore, in consideration of the moderate visible light absorption of these COFs, we also conducted photocatalytic H_2_O_2_ production using 380 nm of LED light as the monochromatic illuminant. This showed H_2_O_2_ photosynthetic rates of 491, 759, 1140 and 3324 μmol g^−1^ h^−1^ for N-COF, S-COF, N-COF* and S-COF*, respectively (Fig. 6a). It is worth noting that S-COF* showed dramatic enhancement in photocatalytic activity, up to four times that of the parent **S-COF**. Although recently, Liao *et al.* reported pyrazine-functionalized COFs with a highest photocatalytic H_2_O_2_ production rate of 7327 μmol g^−1^ h^−1^ in COF photocatalysts [50], the H_2_O_2_ synthesis rates of most reported COFs are quite moderate [46]. Thanks to the activity enhancement resulting from the PCS state, the excellent photocatalytic performance of S-COF* makes it one of the best porous crystalline photocatalysts with regard to H_2_O_2_ production (as listed in Table S1). Cycling stability tests were also carried out to evaluate the durability of the photocatalyst for N-COF* and S-COF*, showing slightly decreased catalytic performance after five cycles of photocatalytic tests (Fig. 6c). These results provide evidence that the PCS states in these COFs were very stable, with a substantially long lifetime.

### Photocatalytic mechanism studies

To investigate the mechanism of photocatalytic H_2_O_2_ production for the above COFs catalysts, a series of control experiments were carried out on N-COF* and S-COF*. With Ar bubbling for 30 min to eliminate O_2_ or in dark, both N-COF* and S-COF* displayed H_2_O_2_ photosynthetic rates less than 30 μmol g^−1^ h^−1^, demonstrating the indispensable process of the photocatalysis of O_2_. Then, photocatalytic water oxidation reaction (WOR) for the H_2_O_2_ production was studied with Ar in AgNO_3_ aqueous solution. N-COF* and S-COF* exhibited photocatalytic activity with H_2_O_2_ photosynthetic rates of 220 and 150 μmol g^−1^ h^−1^, respectively (Fig. 6d), and almost no dioxygen was detected under this condition. These results revealed that both photocatalytic WOR and ORR processes of H_2_O_2_ production existed in these COFs. Moreover, an isotopic experiment to simulate the real system (without AgNO_3_) was conducted for N-COF* and S-COF* to verify the as-proposed mechanism of ORR by using ^18^O_2_ (as an electron acceptor) and H_2_^16^O (as an electron donor). ^18^O_2_ (*m*/*z* = 36) was detected in the decomposition product of the reaction between photogenerated H_2_O_2_ and MnO_2_ (Fig. 6f and Fig. S49), indicating the H_2_O_2_ photosynthesis process from ORR.

As for the H_2_O_2_ photosynthesis reaction mechanism, there are two possible routes for ORR and WOR from O_2_ and water via the 2e^−^ redox process, respectively. One of them is a 2e^−^ two-step process with hydroxyl radicals (^•^OH) and superoxide anion radicals (^•^O_2_^−^) as the intermediate species for indirect H_2_O_2_ generation, and the other is a direct 2e^−^ one-step process. In order to verify the mechanism of the full reaction of H_2_O_2_ photosynthesis for the above two COFs, *in situ* EPR was conducted using 5,5-dimethyl-pyrroline *N*-oxide (DMPO) as the free-radical spin-trapping agent to detect ^•^OH and ^•^O_2_^−^. As depicted in Fig. 6e and Fig. S50, typical characteristic signals of DMPO-^•^OOH and DMPO-^•^OH species were observed in O_2_-saturated water for N-COF* and S-COF* under Xe lamp irradiation and were both absent in the dark condition. This implies that the H_2_O_2_ photosynthesis reaction in this COF system complies with a 2e^−^ two-step process with the participation of ^•^OH and ^•^OOH.

A possible mechanism can be proposed to illustrate the photosynthesis of H_2_O_2_ for the **zyg**-type COFs according to the experimental results. Briefly, with regard to **N-COF**, the dioxygen is adsorbed and gains electrons as well as protons at the TAPB-Me sites to generate ^•^OOH active intermediate. Meanwhile, the H_2_O molecule utilizes the holes from the TPA sites to form ^•^OH active intermediate. Then, ^•^OOH and ^•^OH active intermediates simultaneously produce H_2_O_2_ at **N-COF** and N-COF*. There is a similar situation with **S-COF** and S-COF* except that the hole utilization for the H_2_O molecule happens at PTZ sites instead of TPA sites. In addition, the PCS states in the COF structures result in different enhancements of the photocatalytic H_2_O_2_ production rates.

To further understand the catalytic mechanism and the reason why the catalytic performances are distinct for these COFs, DFT calculations analyzing the contributions of the molecular orbital and the Gibbs free energy of the ORR and WOR processes were conducted. First, the molecular orbital analysis was carried out on the constructed cluster models, revealing that both **N-COF** and **S-COF** present an excellent spatial charge separation in which the HOMO is mainly located in the TPA and PTZ moieties for **N-COF** and **S-COF**, respectively, and the LUMO is dominantly located at the imine and its adjacent phenyl group in the TAPB-Me site (Fig. S51). Thus, we can deduce the reaction pathway as follows: first, during the light activation process of forming the corresponding excited species N-COF* and S-COF* from **N-COF** and **S-COF**, the electron transfer probably takes place from the TPA or PTZ group to the imine group site and produces the metastable PCS states; second, the photocatalytic reduction reaction occurs around the electron-rich positions (imine sites) and the oxidation reaction at the hole positions (the TPA or the PTZ sites) for both of **N-COF** and **S-COF**. Since the excitation process is realized by light irradiation, the metastable radical species after excitation have been captured by experiments. Therefore, the theoretical calculation is based on the N-COF* and S-COF* intermediates after the PCS process. For comparison, **N-COF** and **S-COF** catalytic potential energy surfaces before excitation are also considered (Fig. 6g). In addition, because **N-COF** and **S-COF** contain the same building unit TAPB-Me, the mechanism of the ORR catalytic process is the same for both. So, there are only two lines on the potential energy surface of the ORR process (red line represents the ground state species for **N-COF**/**S-COF** and blue line represents the excited species for N-COF*/S-COF* in Fig. 6g).

In general, the first process in ORR and WOR is the potential determination step for the photocatalytic two-step H_2_O_2_ production. Based on the calculation results, with regard to the ORR process, the O_2_ molecules are adsorbed at the imine N sites and then experience one electron reduction and protonation to form ^•^OOH intermediate. The Gibbs free energy change (Δ*G*^o^) values for this process are −2.23 eV for **N-COF**/**S-COF** and −4.63 eV for N-COF*/S-COF*, meaning that the ORR is thermodynamically favorable for all these COFs and more energy-favorable for the excited species N-COF*/S-COF*. Then one more photogenerated electron and proton transfer into the ^•^OOH intermediate, fulfilling the two-step ORR process from O_2_ to H_2_O_2_ product with Δ*G*^o^ values of −3.23 eV and −5.72 eV for **N-COF**/**S-COF** and N-COF*/S-COF* relative to the initial point, respectively. This reveals that the electron-withdrawing status (reductive TAPB-Me species) for **N-COF** and **S-COF** is more beneficial to the photocatalytic ORR process. With regard to the WOR process, the H_2_O molecules are adsorbed at the N atom of TPA for **N-COF**/N-COF* and at the phenothiazine ring for **S-COF**/S-COF*, and one electron oxidation as well as deprotonation subsequently occurs, resulting in the generation of ^•^OH intermediate. The Δ*G*^o^ values of this step are 2.69 eV and 2.63 eV for **N-COF** and N-COF*, respectively, which are comparable before and after light activation. By contrast, the free energies of forming ^•^OH intermediate show the distinct energy difference for **S-COF** and S-COF* (Δ*G*^o^ = 2.32 eV and 0.88 eV), respectively. Two ^•^OH intermediates would desorb and combine to generate H_2_O_2_, achieving the two-step WOR process with Δ*G*^o^ values of 1.19, 1.14, 1.57 and 0.14 eV for N-COF, N-COF*, S-COF and S-COF* (Fig. 6g), respectively. These results reveal that the electron-donating status is beneficial to the photocatalytic WOR process for **S-COF** (oxidized PTZ species) while it has little effect on **N-COF** (oxidized TPA species). These results explain the catalytic performance differences as below: (i) the photocatalytic H_2_O_2_ production performance was comparable for the ground-states **N-COF** and **S-COF**; (ii) the photocatalytic H_2_O_2_ production performance was enhanced for the excited-states N-COF*/S-COF* compared with **N-COF**/**S-COF**; (iii) the performance enhancement compared to their initial states was much more significant for S-COF* than N-COF*.

## CONCLUSION

In summary, we have demonstrated a design strategy of utilizing inequilateral hexagonal building units to prepare 3D COFs (**M-COF, N-COF, S-COF** and **T-COF**) based on a [6 + 4] network with a previously unreported **zyg** topology in COF structures. It is noteworthy that these **zyg**-type COFs exhibit two types of 1D channel and a fascinating fused Olympic-rings-like pore shape in the unit cell *c* axis. Both **N-COF** and **S-COF**, containing photocatalytically active groups, showed excellent activity in the photosynthesis of H_2_O_2_ compared to other COF photocatalysts. Subsequently, the photochromic radical states for the above two COFs (N-COF* and S-COF*) were successfully obtained, showing dramatic enhancement of photoactivity for H_2_O_2_ synthesis compared to the parent materials. Remarkably, S-COF* showed a H_2_O_2_ photosynthetic rate of 3324 μmol g^−1^ h^−1^, >400% enhancement in activity compared to **S-COF**, making S-COF* one of the best porous crystalline photocatalysts in H_2_O_2_ photosynthesis. This work provides an effective strategy for constructing PRSs in **zyg**-type 3D COFs, which facilitate significant photocatalytic activity enhancement for H_2_O_2_ synthesis. This work will shed light on the development of efficient 3D COF photocatalysts, building on the functional organic building blocks assembled in topologies to facilitate photoinduced charge separation.

## Supplementary Material

nwae177_Supplemental_Files
