# Supplementary material for: Photochromic radical states in 3D covalent organic frameworks with zyg topology for enhanced photocatalysis
Source: Natl Sci Rev. 2024 May 21;11(7):nwae177. doi: 10.1093/nsr/nwae177 (PMC11173181; doi:10.1093/nsr/nwae177)
Supplement: nwae177_Supplemental_Files [file nwae177_supplemental_files.zip › Supplementary Information.docx]

**[Supplementary Information]**

**Photochromic Radical States in Three-Dimensional Covalent Organic Frameworks with zyg Topology for Enhanced Photocatalysis**

Tian-Tian Ma,^1,†^ Guo-Zhang Huang,^2,†^ Xiao-Han Wang,^1,†^ Yan Liang,^1^ Run-Han Li,^1^ Bin Wang,^1^ Su-Juan Yao,^1^ Jia-Peng Liao,^1^ Shun-Li Li,^1^ Yong Yan,^1,^* and Ya-Qian Lan^1,^*

*1 School of Chemistry, South China Normal University, Guangzhou, Guangdong 510006, China.*

*2 Department of Chemistry, Guangdong Provincial Key Laboratory of Catalytic Chemistry, Southern University of Science and Technology, Shenzhen, Guangdong 518055, China.*

*†Equally contributed to this work.*

**Corresponding authors*

*Y. Y. E-mail:* *yong.yan@m.scnu.edu.cn*

*Y.-Q. L. E-mail: yqlan@m.scnu.edu.cn*

**Contents**

[S1. Experimental Section 3](#_Toc166099510)

[S1.1. Materials, characterization and synthetic procedures 3](#_Toc166099511)

[S1.2. Chemical stability tests 9](#_Toc166099512)

[S1.3. Solid state ^13^C cross-polarization magic angle spinning NMR 10](#_Toc166099513)

[S1.4. Digestion experiments for COFs 14](#_Toc166099514)

[S1.5. Fourier-transform infrared spectroscopy 16](#_Toc166099515)

[S1.6. Thermogravimetric analysis 18](#_Toc166099516)

[S1.7. Scanning electron microscopy 19](#_Toc166099517)

[S1.8. PXRD patterns and Rietveld refinement results for COFs 20](#_Toc166099518)

[S1.9. Structure simulations and X-ray diffraction analyses 23](#_Toc166099519)

[S1.10. Transmission electron microscopy and MicroED 28](#_Toc166099520)

[S1.11. Gas sorption data 31](#_Toc166099521)

[S1.12. Electron paramagnetic resonance 33](#_Toc166099522)

[S2. Photocatalytic Experiments and Photoelectrochemical Measurements 35](#_Toc166099523)

[S2.1. Photocatalytic experiments and characterizations 35](#_Toc166099524)

[S2.2. Mott-Schottky measurements 37](#_Toc166099525)

[S2.3. Transient photocurrent response 39](#_Toc166099526)

[S2.4. Stability tests 40](#_Toc166099527)

[S2.5. H_2_O_2_ detection methods 42](#_Toc166099528)

[S2.6. Isotope calibration 44](#_Toc166099529)

[S2.7. *In-situ* electron paramagnetic resonance 45](#_Toc166099530)

[S2.8. DFT calculation 46](#_Toc166099531)

[S3. Crystallographic Information 48](#_Toc166099532)

# S1. Experimental Section

## S1.1. Materials, characterization and synthetic procedures

**Materials**

All solvents and reagents obtained from commercial sources were used without further purification. 1, 2-dichlorobenzene, dichloromethane, 1, 4-dioxane, acetone, toluene, *N*, *N*-dimethylformamide (DMF, AR), *N*, *N*-dimethylacetamide (DMA, AR), tetrahydrofuran (THF, 99.5%), trifluoroacetic acid (TFA, AR) were purchased from Sinopharm Chemical Reagent Co., Ltd. 1,3,5-Tribromobenzene, tris (4-bromophenyl) amine, 1,3,5-tris(4-bromophenyl)benzene were purchased from Adamas. (3,5-Bis(5,5-dimethyl-1,3-dioxan-2-yl)phenyl)boronic acid, 3,7-dibromo-10-(4-bromophenyl)-10H-phenothiazine, 1,2,4,5-tetrakis-(4-aminophenyl)-3',6'-dimethylbenzene were purchased from Shanghai Tensus Biotech CO., Ltd. 5-(4,4,5,5-Tetramethyl-1,3,2-dioxaborolan-2-yl)-1,3-benzenedicarboxaldehyde was purchased from Leyan Chemical Reagent Co., Ltd..

**Characterization**

The powder X-ray diffraction (PXRD) spectra were recorded on a Bruker D8 Advance diffractometer (Cu Kαradiation, λ = 1.54060 Å, at 40 kV, 40 mA). Diffraction intensity data for 2θ from 1.5 ~ 50° were collected at the scanning speed of 2 deg./min with 2θ step increment of 0.01°. Fourier-transform infrared (FT-IR) spectra of starting materials and COF samples were recorded on a Bruker (ALPHA) spectrometer in the range of 4000-400 cm^-1^ using the KBr pellets. ^13^C CP/MAS solid-state nuclear magnetic resonance (SSNMR) spectra were obtained from JEOL 600M spectrometer equipped with a 14.09 T superconducting magnet and a 4 mm double-resonance MAS probe (JEOL RESONANCE Inc., Japan). The surface morphology of samples was collected using a high-resolution thermal field emission scanning electron microscope (SEM, TESCAN MIRA LMS) with an acceleration voltage of 15 kV. Transmission electron microscopy (TEM) images were obtained on a FEI-Talos F200S microscopy at an accelerating voltage of 200 kV. The UV-vis absorption spectra of COFs were recorded on an Agilent Cary 5000 spectrophotometer. Electron diffraction patterns were taken by a JEM-2100 Plus transmission electron microscope. Nitrogen sorption isotherms was measured at 77 K using an Autosorb IQ2 absorptiometer (Quantachrome Instruments) volumetric adsorption analyzer. Apparent surface areas were determined using the BET method. Pore size distributions of all materials were calculated using the nonlocal density functional theory (NL-DFT) model. Thermogravimetric (TGA) analysis of COFs powder samples was performed on a NETZSCH TG 209F1 Libra Thermal Analyzer System with heating rate of 10 °C min^-1^ to 800 °C under N_2_ atmosphere, respectively. A UV LED lamp (365-405 nm, 18 W) was used to simulate light radiation at room temperature. The distance between then lamp and samples was set as 10 cm. Electron paramagnetic resonance (EPR) data were achieved in the X band (frequency of micro-wave, 9.83 GHz) at room temperature on a Bruker Model A300 spectrometer with a 100 kHz magnetic field. The C, H and N contents in the COF sample were determined using an Elementar Vario-EL CHNS elemental analyzer.

**Synthesis of monomers**

**Ph-6CHO**

***5'-(3,5-diformylphenyl)-[1,1':3',1''-terphenyl]-3,3'',5,5''-tetracarbaldehyde***

***
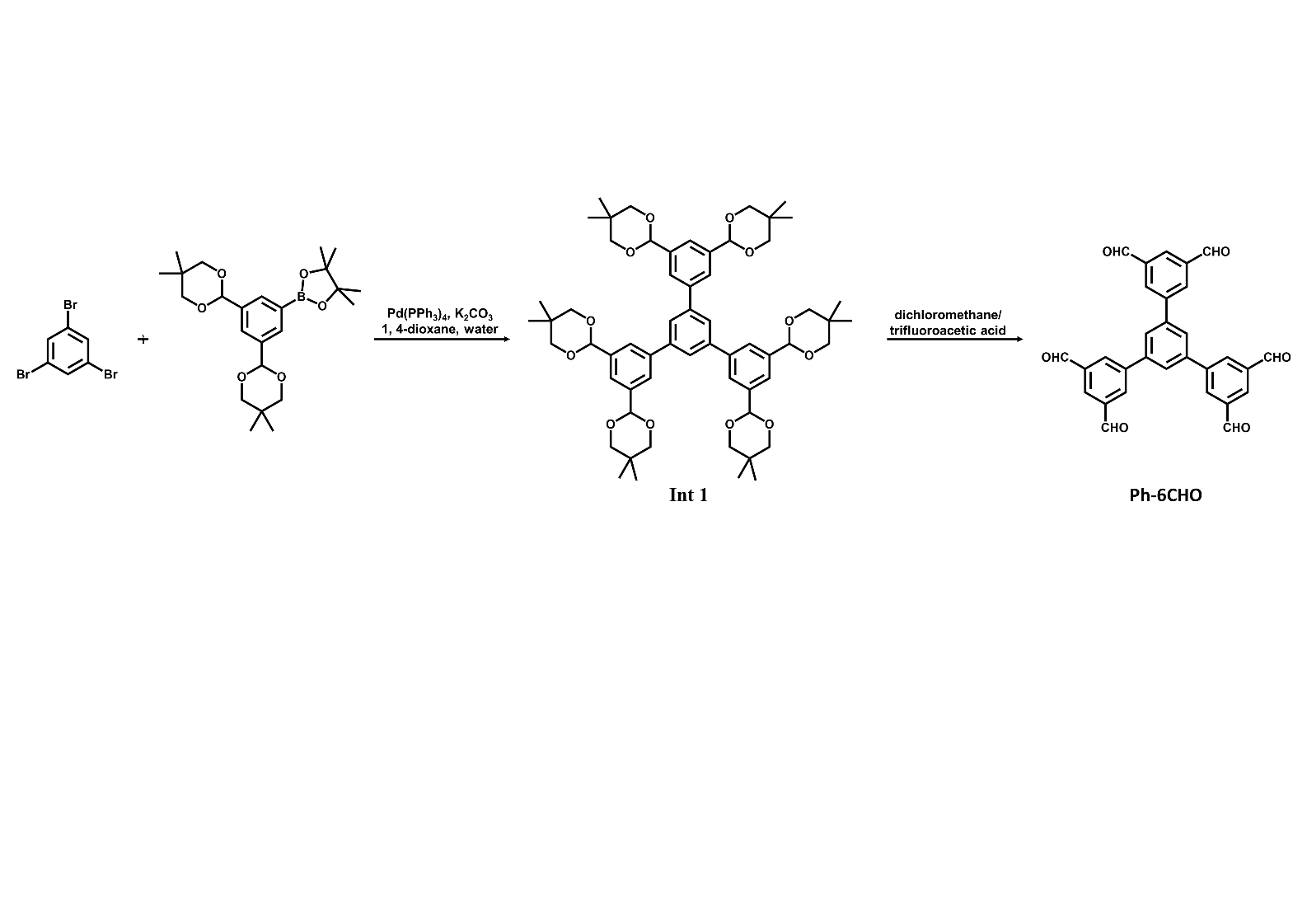
***

A mixture of 1,3,5-tribromobenzene (3.14 g, 10 mmol), (3,5-bis(5,5-dimethyl-1,3-dioxan-2-yl)phenyl)boronic acid (15.76 g, 45 mmol), K_2_CO_3_ (6.21 g, 45 mmol) and Pd(PPh_3_)_4_ (1.16 g, 1 mmol) were charged in a three-neck round bottom flask containing 1, 4-dioxane/water (120/30 ml). The mixture was purged with nitrogen for 30 minutes and heated under nitrogen at 110 °C for two days. After cooling to room temperature, the organic solvent was evaporated under reduced pressure and the residue was diluted with H_2_O (50 mL), extracted with dichloromethane (three times, 80 mL per time). The combined organic layer was washed with brine, dried over Na_2_SO_4_, and then evaporated to dryness. The crude product was purified by flash column chromatography on silica gel to give the corresponding product (**Int 1**) (off-white solid, 83% yield). To a stirred mixture of **Int 1** (8.06 g, 7.5 mmol) in dichloromethane (100 mL) was added trifluoroacetic acid at 0 ℃. After being stirred for 0.5 h at 0 ℃, the mixture was stirred for 10 h at ambient temperature. The reaction mixture was filtered and the filtered cake washed with purified water and EtOH for three times (5 mL per time). The product obtained was extremely poorly soluble and almost insoluble in all common organic solvents. (off-white solid, 94% yield). **Int 1:** ^1^H NMR (600 MHz, Chloroform-*d*): δ 7.78 (s, 9H), 7.66 (s, 3H), 5.48 (s, 6H), 3.79 (d, *J* = 11.3 Hz, 12H), 3.68 (s, 12H), 1.30 (s, 18H), 0.80 (s, 18H). ^13^C NMR (151 MHz, Chloroform-*d*): δ 142.13, 141.67, 139.26, 125.91, 123.22, 101.65, 77.79, 30.42, 23.38, 22.09.

**TPA-6CHO**

***4',4''',4'''''-nitrilotris(([1,1'-biphenyl]-3,5-dicarbaldehyde))***

***
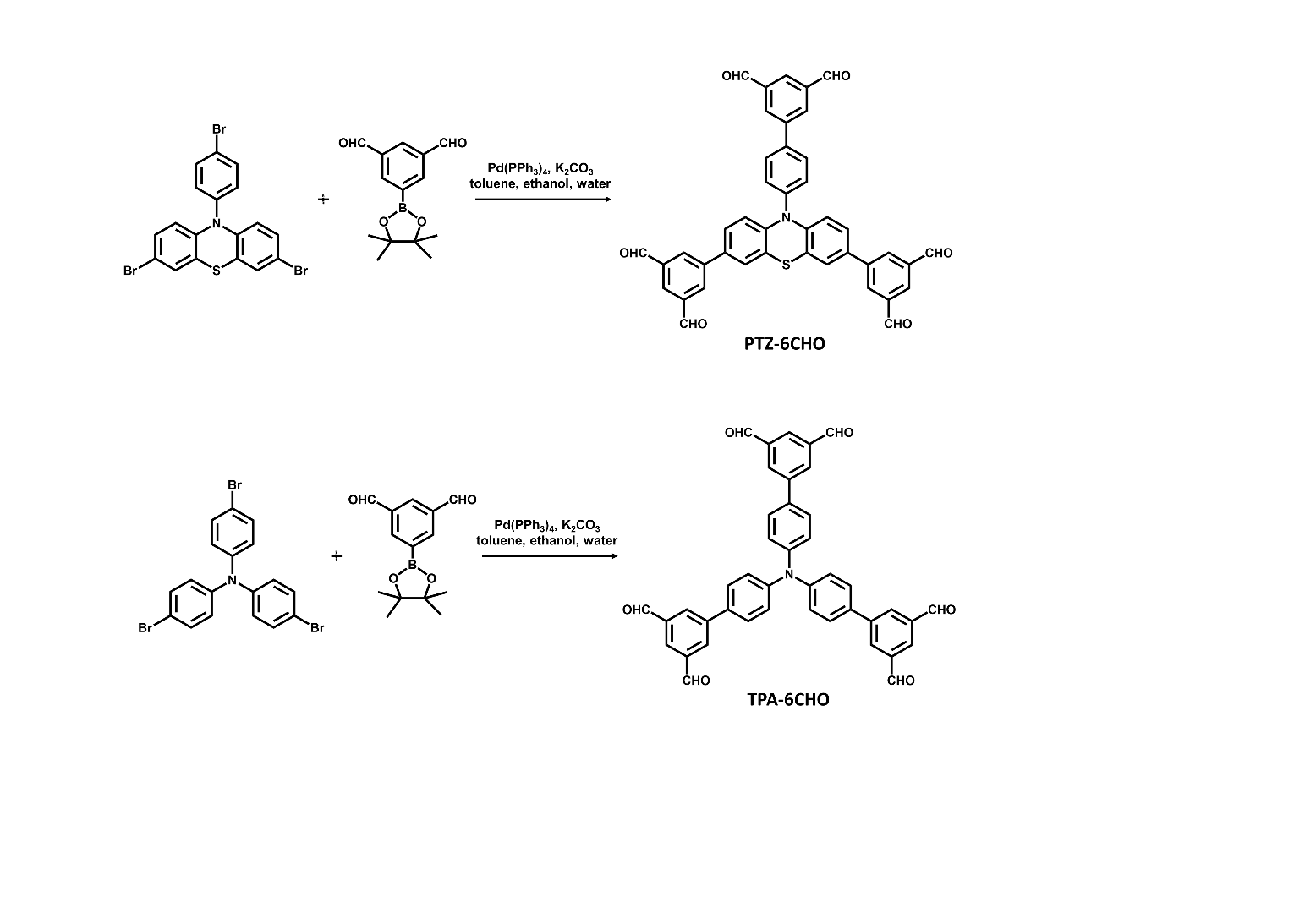
***

TPA-6CHO was synthesized according to a previously published procedure with slight modification.^1^ Detailly, a mixture of tris(4-bromophenyl)amine (1.25 g, 2.6 mmol), 5-(4,4,5,5-tetramethyl-1,3,2-dioxaborolan-2-yl)-1,3-benzenedicarboxaldehyde (2.42 g, 9.3 mmol), Pd(PPh_3_)_4_ (300 mg, 0.26 mmol) and K_2_CO_3_ (7.17 g, 51.9 mmol) were charged in a three-neck round bottom flack containing toluene/ethanol/water (120/40/26 mL). The mixture was purged with nitrogen for 30 minutes and heated under nitrogen at 95 °C for 3 days. After cooling to room temperature, the solvent was removed under reduced pressure. The residue was washed with water, ethanol and hexane sequentially for three times to afford a yellowish-green crude product The residue was passed through a flash silica gel column with acetone-dichloromethane (V/V, 10:1) as eluent to afford the pure TPA-6CHO linker. (yellow solid, 65% yield). ^1^H NMR (600 MHz, DMSO-*d*_6_): δ 10.19 (s, 6H), 8.51 (d, *J* = 1.5 Hz, 6H), 8.36 (s, 3H), 7.86 (d, *J* = 8.7 Hz, 6H), 7.29 (d, *J* = 8.7 Hz, 6H). ^13^C NMR (151 MHz, DMSO-*d*_6_) δ 193.21, 147.52, 141.66, 137.95, 132.93, 128.79, 128.16, 125.01.

**PTZ-6CHO**

***5,5'-(10-(3',5'-diformyl-[1,1'-biphenyl]-4-yl)-10H-phenothiazine-3,7 diyl)diisophthalaldehyde***

***
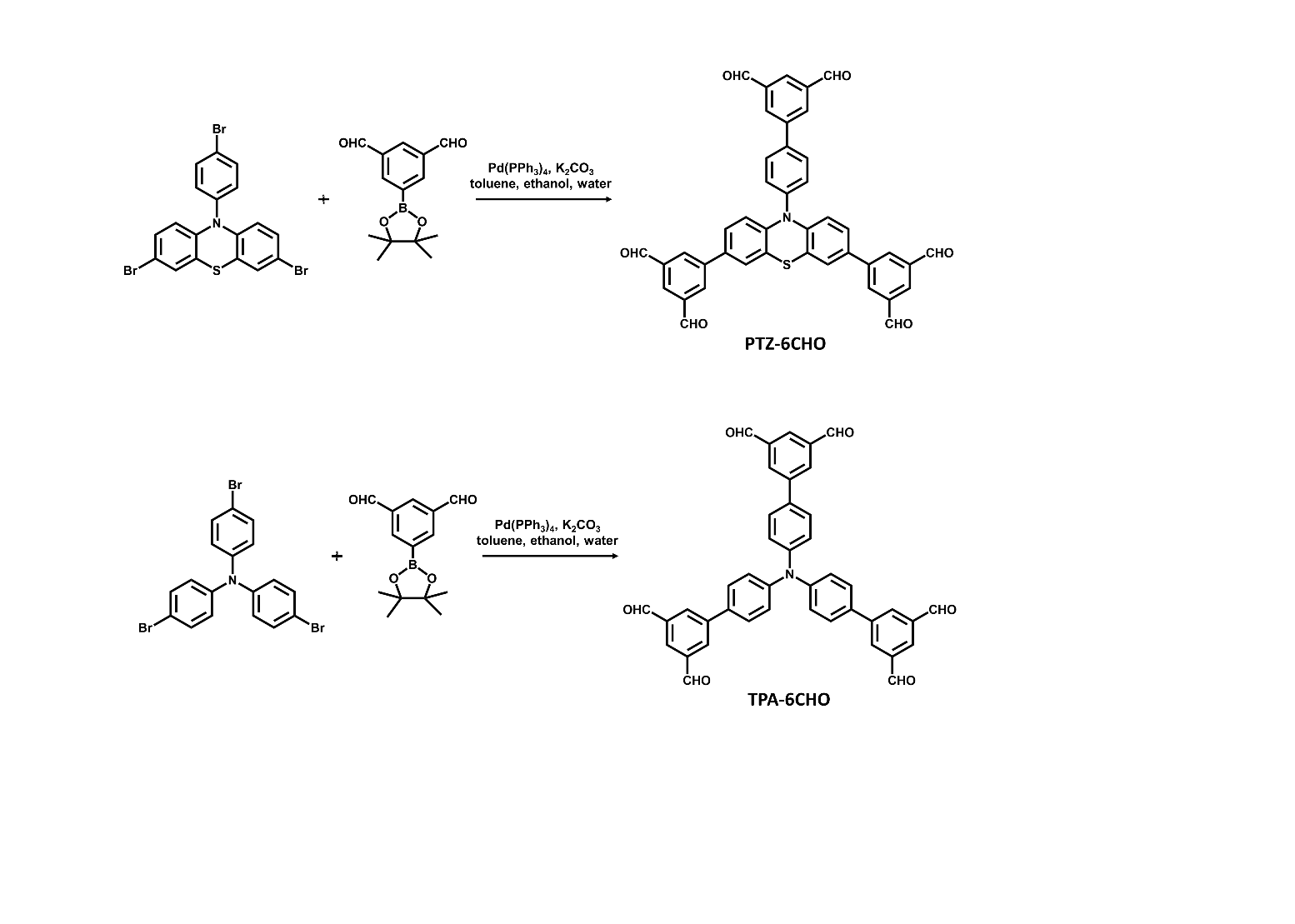
***

A mixture of 3,7-dibromo-10-(4-bromophenyl)-10H-phenothiazine (900 mg, 1.74 mmol), 5-(4,4,5,5-tetramethyl-1,3,2-dioxaborolan-2-yl)-1,3-benzenedicarboxaldehyde (1.9 g, 7.3 mmol), Pd(PPh_3_)_4_ (201 mg, 0.17 mmol) and K_2_CO_3_ (4.82 g, 34.9 mmol) were charged in a three-neck round bottom flask containing toluene/ethanol/water (80/30/20 mL). The mixture was purged with nitrogen for 30 minutes and heated under nitrogen at 95 °C for 3 days. After cooling to room temperature, the solvent was removed under reduced pressure. The residue was washed with water, ethanol and hexane in turn to afford a brownish-red solid. The residue was passed through a flash silica gel column with acetone/dichloromethane (V/V, 10:1) as eluent to afford PTZ-6CHO as an orange-red solid. (72% yield)). ^1^H NMR (600 MHz, DMSO-*d*_6_): δ 10.22 (s, 2H), 10.14 (s, 4H), 8.63 (s, 2H), 8.48 – 8.39 (m, 5H), 8.32 – 8.27 (m, 2H), 8.18 (d, *J* = 7.9 Hz, 2H), 7.69 (d, *J* = 7.9 Hz, 2H), 7.59 (d, *J* = 2.0 Hz, 2H), 7.41 (d, *J* = 8.5 Hz, 2H), 6.33 (d, *J* = 8.5 Hz, 2H). ^13^C NMR (151 MHz, DMSO-*d*_6_): δ 193.07, 143.54, 141.19, 140.72, 140.66, 138.62, 137.99, 137.83, 133.71, 132.80, 132.61, 131.69, 130.40, 128.78, 128.15, 126.43, 125.41, 120.41, 116.95.

**TPB-6CHO**

***5''-(3',5'-diformyl-[1,1'-biphenyl]-4-yl)-[1,1':4',1'':3'',1''':4''',1''''-quinquephenyl]-3,3'''',5,5''''-tetracarbaldehyde***

**
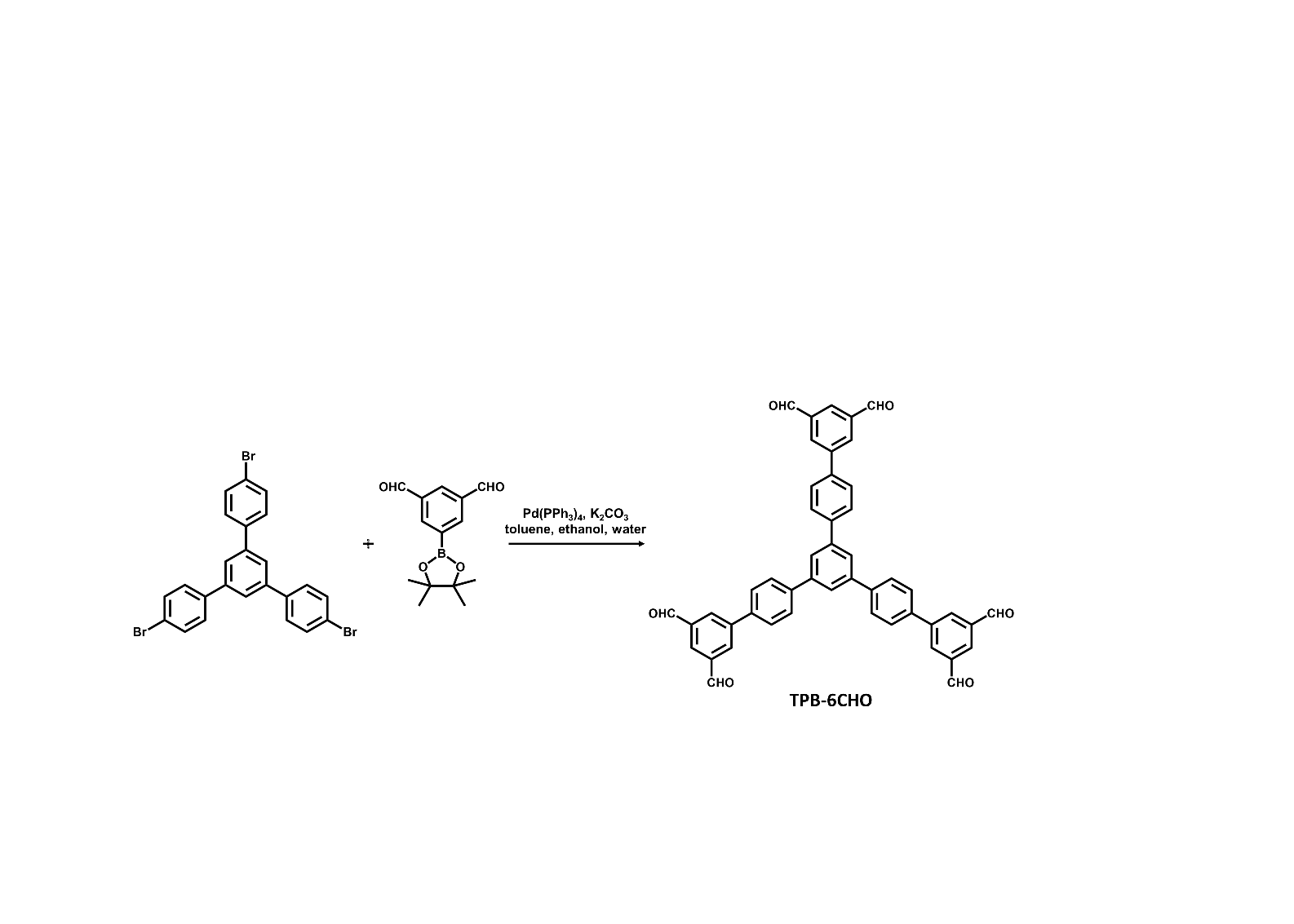
**

A mixture of 1,3,5-tris(4-bromophenyl)benzene (1.3 g, 2.4 mmol), 5-(4,4,5,5-tetramethyl-1,3,2-dioxaborolan-2-yl)-1,3-benzenedicarboxaldehyde (2.5 g, 9.6 mmol), Pd(PPh_3_)_4_ (277 mg, 0.24 mmol) and K_2_CO_3_ (6.6 g, 47.8 mmol) were charged in a three-neck round bottom flask containing toluene/ethanol/water (120/40/24 mL). The mixture was purged with nitrogen for 30 minutes and heated under nitrogen at 95 °C for 3 days. After cooling to room temperature, the solvent was removed under reduced pressure. The light-grey crude product was washed with water, ethanol and dichloromethane in turn to afford TPB-6CHO as an off-white solid (70% yield). ^1^H NMR (600 MHz, DMSO-*d*_6_): δ 10.24 (s, 6H), 8.63 (d, *J* = 1.5 Hz, 6H), 8.43 (t, *J* = 1.4 Hz, 3H), 8.16 – 8.13 (m, 6H), 8.12 (s, 3H), 8.05 – 8.01 (m, 6H). ^13^C NMR (151 MHz, DMSO-*d*_6_): δ 193.23, 141.82, 141.47, 140.67, 138.00, 137.58, 133.43, 128.61, 128.49, 128.01, 125.21.

**Synthesis of COFs**

**M-COF**

A Pyrex tube (Beijing Synthware Glass) was charged with Ph-6CHO (14.4 mg, 0.03 mmol), TAPB-Me (21.3 mg, 0.045 mmol), *o*-DCB (1.5 mL), DCM (1.5 mL), and TFA (60 μL). The mixture was sonicated for 20 minutes and then degassed by three freezing pump-thaw cycles before the tube was sealed and heated at 120°C for 3 days. The off-white precipitate was collected by filtration. The powder was transferred to a Soxhlet extractor and washed with THF for 72 hours. Finally, the product was evacuated under vacuum at 120 °C overnight. Elementary analysis (EA) results: calcd for C_78_H_51_N_6_·10.25H_2_O: C 74.53%, H 5.73%, N 6.69%. Found: C 73.77%, H 4.96%, N 6.19%.

**N-COF**

A Pyrex tube (Beijing Synthware Glass) was charged with TPA-6CHO (19.2 mg, 0.03 mmol), TAPB-Me (21.3 mg, 0.045 mmol), o-DCB (1.5 mL), DCM (1.5 mL), and TFA (60 μL). The same procedure as the synthesis of **M-COF** was used to afford **N-COF** as a yellow powder. EA results: calcd for C_90_H_60_N_7_·10.75H_2_O: C 75.43%, H 5.73%, N 6.84%. Found: C 74.84%, H 4.95%, N 6.28%.

**S-COF**

A Pyrex tube (Beijing Synthware Glass) was charged with PTZ-6CHO (20.4 mg, 0.03 mmol), TAPB-Me (21.3 mg, 0.045 mmol), o-DCB (1.5 mL), DCM (1.5 mL), TFA(90 μL). The same procedure as the synthesis of **M-COF** was used to afford **S-COF** as an orange powder. EA results: calcd for C_90_H_58_N_7_S·11.25H_2_O: C 73.42%, H 5.51%, N 6.66%，S 2.18%. Found: C 73.11%, H 5.24%, N 5.95%, S 1.85%.

**T-COF**

A Pyrex tube (Beijing Synthware Glass) was charged with TPB-6CHO (21 mg, 0.03 mmol), TAPB-Me (21.3 mg, 0.045 mmol), o-DCB (1.5 mL), DCM (1.5 mL), TFA (150 μL). The same procedure as the synthesis of **M-COF** was used to afford **T-COF** as a tan powder. EA results: calcd for C_96_H_63_N_6_·13.5H_2_O: C 74.69%, H5.88%, N 5.44%. Found: C 73.83%, H 4.85%, N 4.80%.

**Photo-activation of the above COFs**

A 50 ml glass tube (Beijing Synthware Glass) was charged with COF samples (30 mg), deionized water (13.5 mL) and isopropyl alcohol (1.5 mL) at 25 ºC under O_2_ atmosphere. Then, the system was irradiated with a xenon lamp source (PLS-SXE300+, Beijing Perfect light). After light irradiation for 8 h, the precipitate was separated by filtration through a filter paper in a Buchner funnel and washed several times with water, acetone and THF. Finally, the product was evacuated under vacuum at 120 °C overnight.

## S1.2. Chemical stability tests

**

**

**Figure S1.** PXRD profiles for pristine **N-COF** and samples after treatments in different solvents.





**Figure S2**. PXRD profiles for pristine **S-COF** and samples after treatments in different solvents.

## S1.3. Solid state ^13^C cross-polarization magic angle spinning NMR


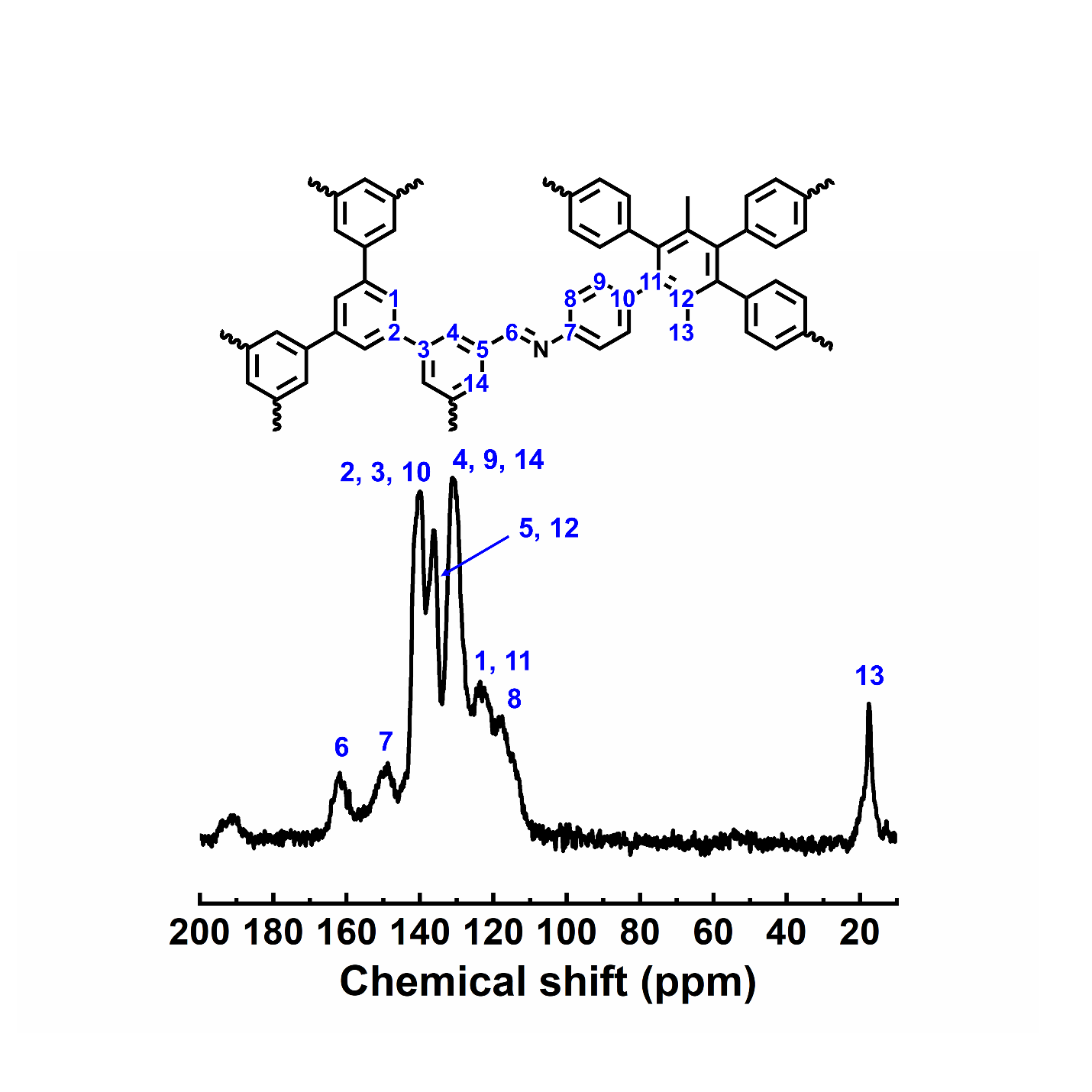


**Figure S3.** Solid-state ^13^C CP/MAS NMR spectrum of **M-COF**.

**
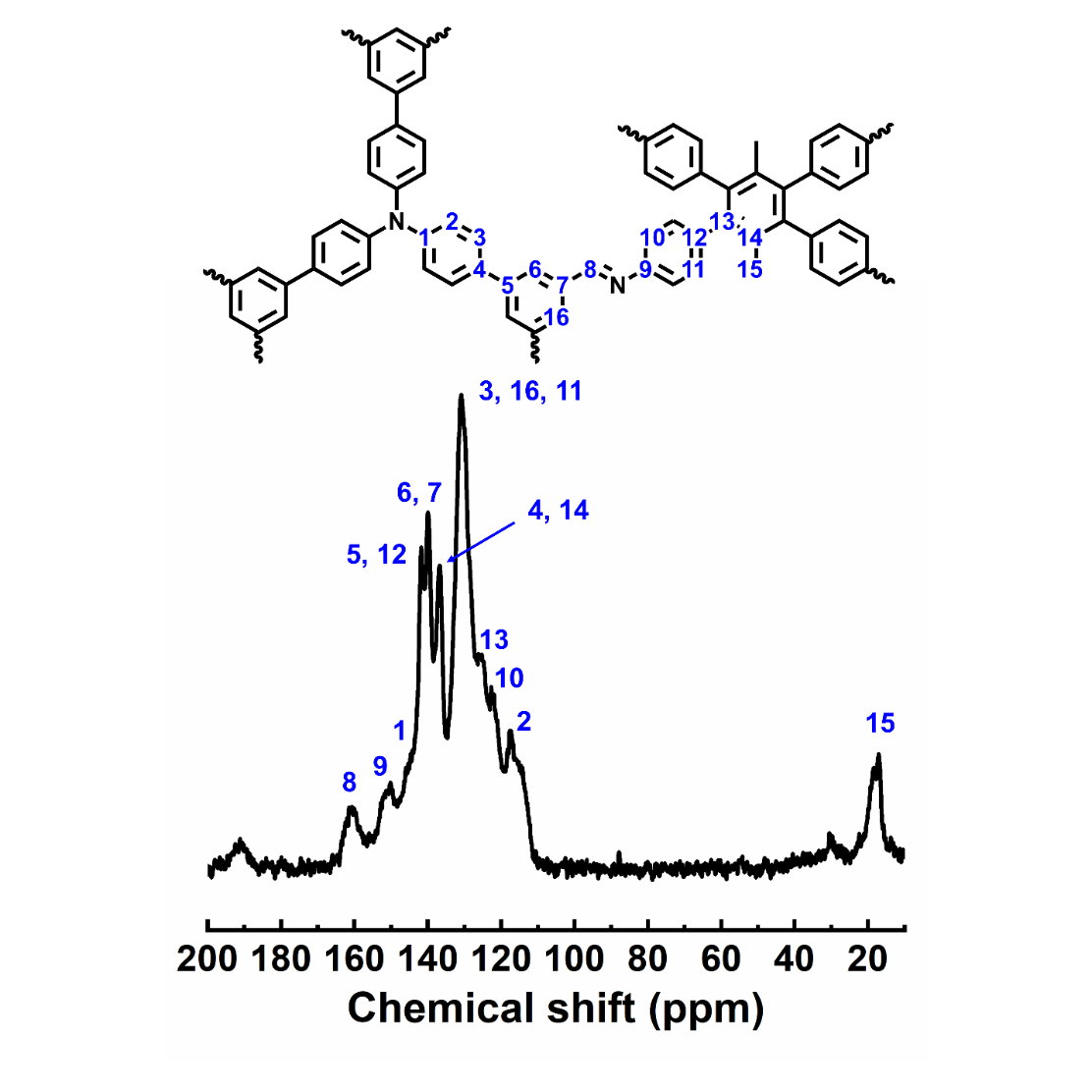
**

**Figure S4.** Solid-state ^13^C CP/MAS NMR spectrum of **N-COF**.

**
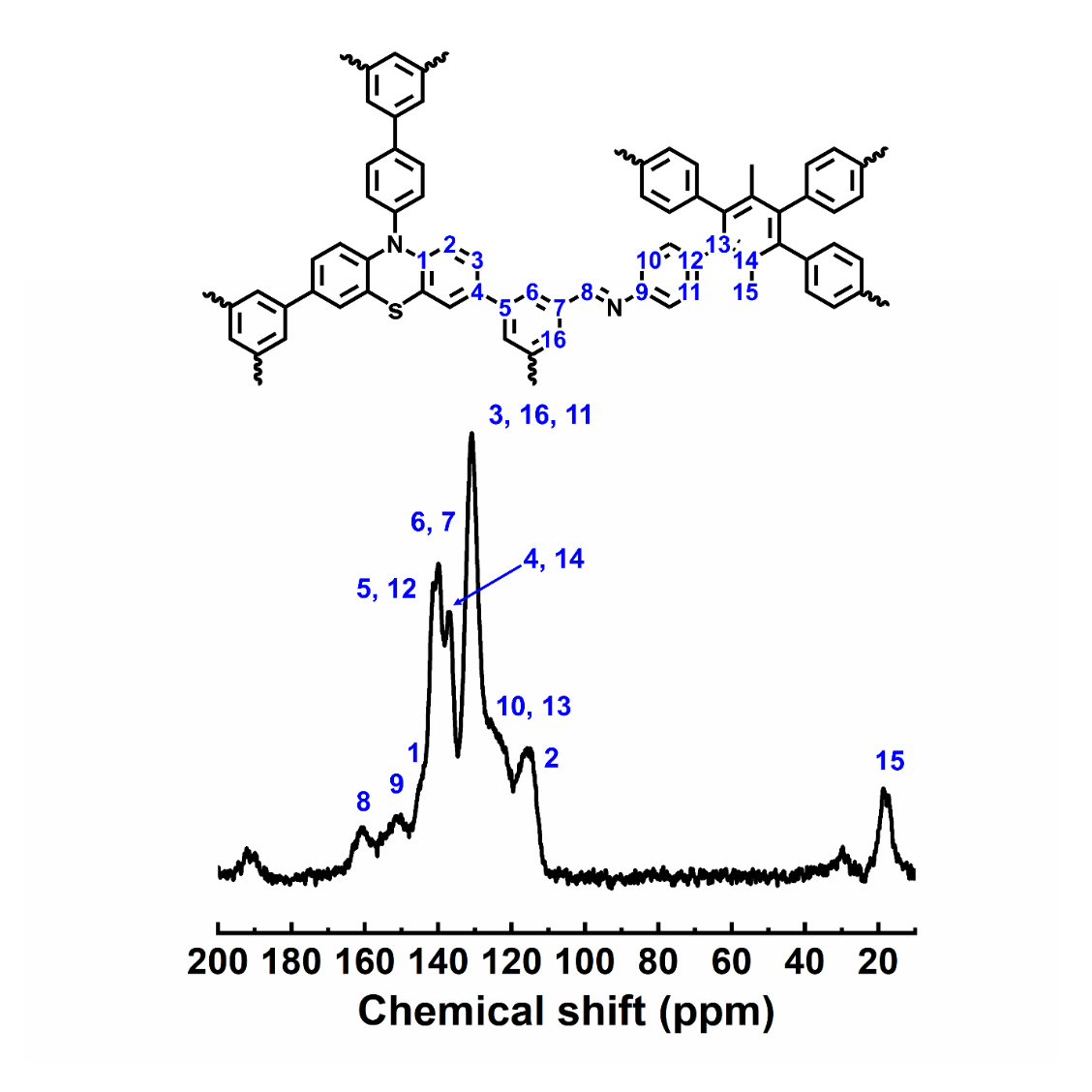
**

**Figure S5.** Solid-state ^13^C CP/MAS NMR spectrum of **S-COF**.

**
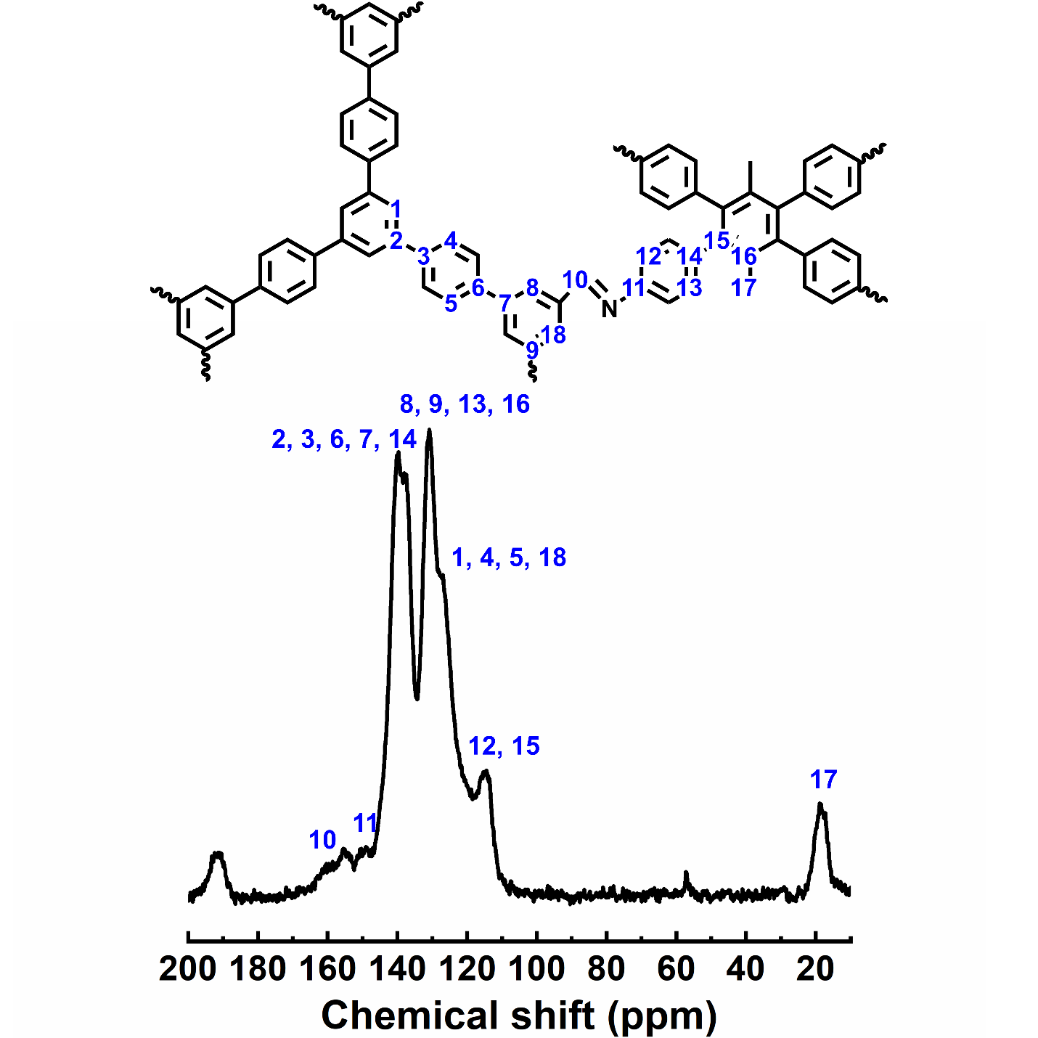
**

**Figure S6.** Solid-state ^13^C CP/MAS NMR spectrum of **T-COF**.

## S1.4. Digestion experiments for COFs

In general, 5 mg of COF was digested with 0.5 mL of DMSO-*d*_6_ and 0.05 mL of DCl (20% in D_2_O). The mixture was sonicated for 5 minutes to fully dissolve the COF.


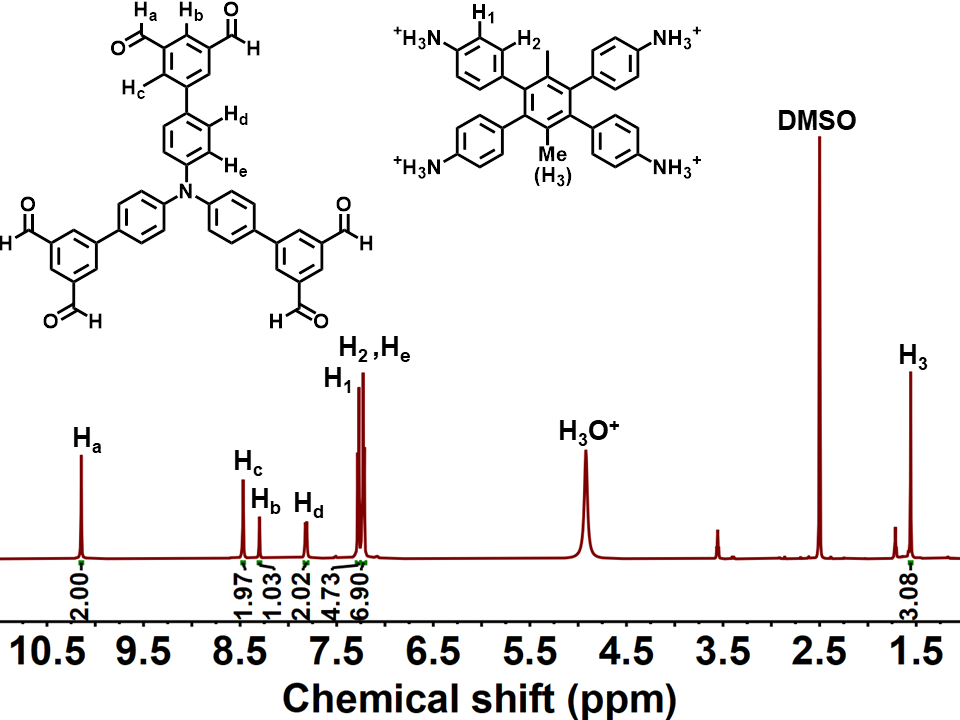


**Figure S7.** ^1^H-NMR spectrum of the acid-digested **N-COF** indicates a linker ratio of **TAPB-Me**/**TPA-6CHO** as 3:2.


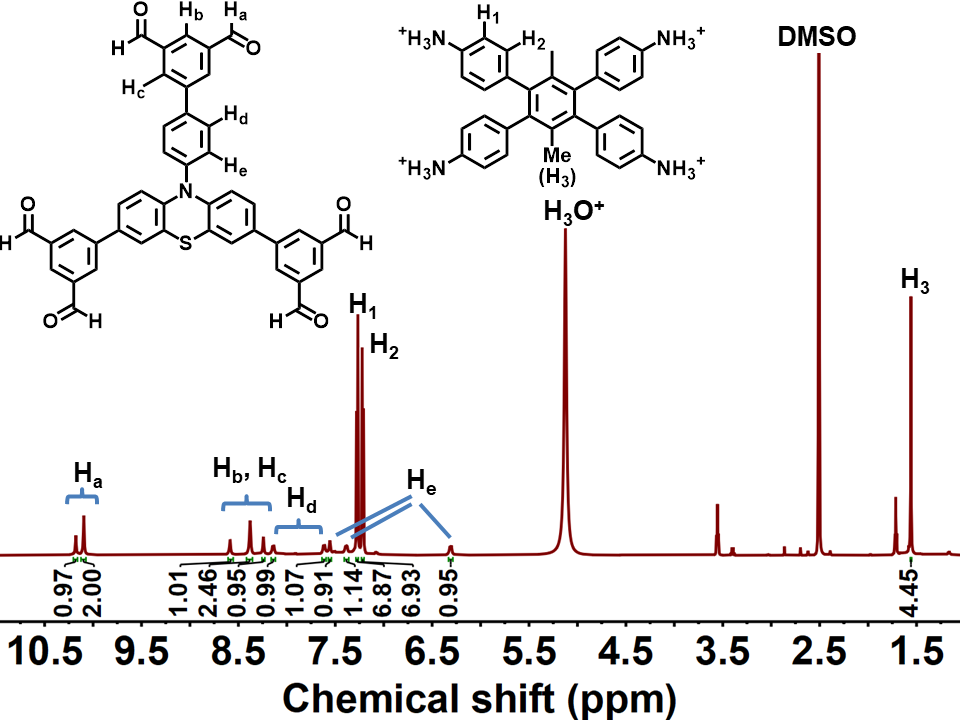


**Figure S8.** ^1^H-NMR spectrum of the acid digested **S-COF** indicates a linker ratio of **TAPB-Me**/**PTZ-6CHO** as 3:2.

## S1.5. Fourier-transform infrared spectroscopy





**Figure S9.** Overlay of FT-IR spectra of activated **M-COF** with starting materials TAPB-Me and Ph-6CHO.





**Figure S10.** Overlay of FT-IR spectra of activated **N-COF** with starting materials TAPB-Me and TPA-6CHO.





**Figure S11.** Overlay of FT-IR spectra of activated **S-COF** with starting materials TAPB-Me and PTZ-6CHO.





**Figure S12.** Overlay of FT-IR spectra of activated **T-COF** with starting materials TAPB-Me and TPB-6CHO.

## S1.6. Thermogravimetric analysis

**

**

**Figure S13.** TGA for **M-COF**, **S-COF**, **N-COF** and **T-COF** under N_2_ atmosphere.

## S1.7. Scanning electron microscopy

**
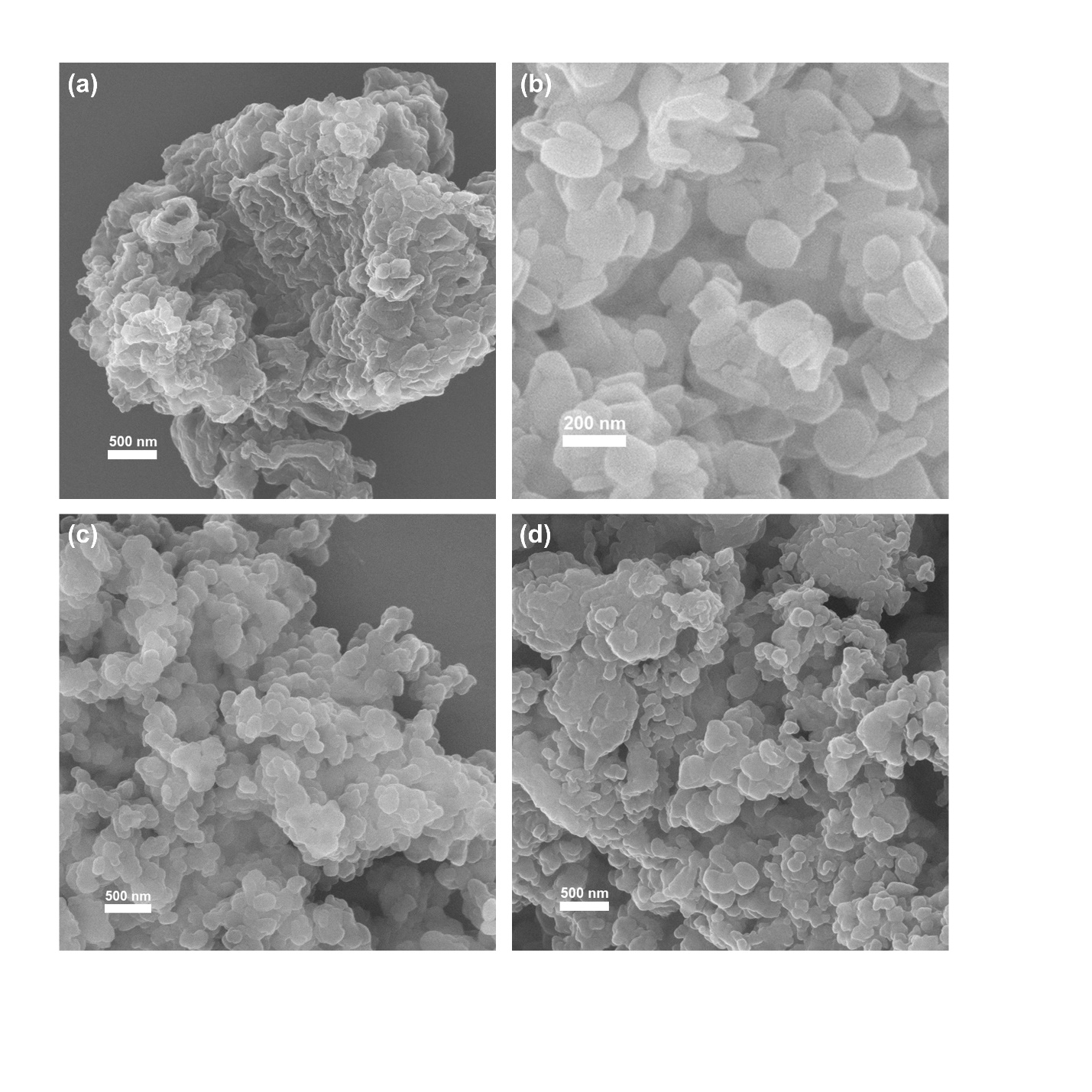
**

**Figure S14.** SEM images of **M-COF** (a), **N-COF** (b), **S-COF** (c) and **T-COF** (d).

## S1.8. PXRD patterns and Rietveld refinement results for COFs

Rietveld analyses were conducted to refine the COF structure models against the experimental PXRD patterns.^2^ Due to the small number of peaks observed in the PXRD patterns for these COFs, the data at high 2θ angles (ie, >20° for **M-COF**; >10° for **N-COF**, **S-COF** and **T-COF**) were ignored in the analysis.

In the refinement processes, the background was modeled by Chebychev polynomials (with terms in range of 6-12, according to different PXRD patterns) and the lattice parameters for each COF were refined as constrained by the hexagonal symmetry. The peak profile shape of the XRPD patterns was described by applying the pseudo-Voigt description. The peak broadening was modeled with asymmetry adopted phenomenological model for microstrain. The axial and zero-error corrections were used, with additional corrections for Gaussian and Lorentzian crystallite size and strain broadening. Rigid bodies were applied to the organic moieties in the framework during refinements. The Rietveld fittings for all these COFs show satisfactory agreement factors.





**Figure S15.** PXRD patterns and refinement result for **M-COF**.





**Figure S16.** PXRD patterns and refinement result for **N-COF**.





**Figure S17.** PXRD patterns and refinement result for **S-COF**.





**Figure S18.** PXRD patterns and refinement result for **T-COF**.

## S1.9. Structure simulations and X-ray diffraction analyses


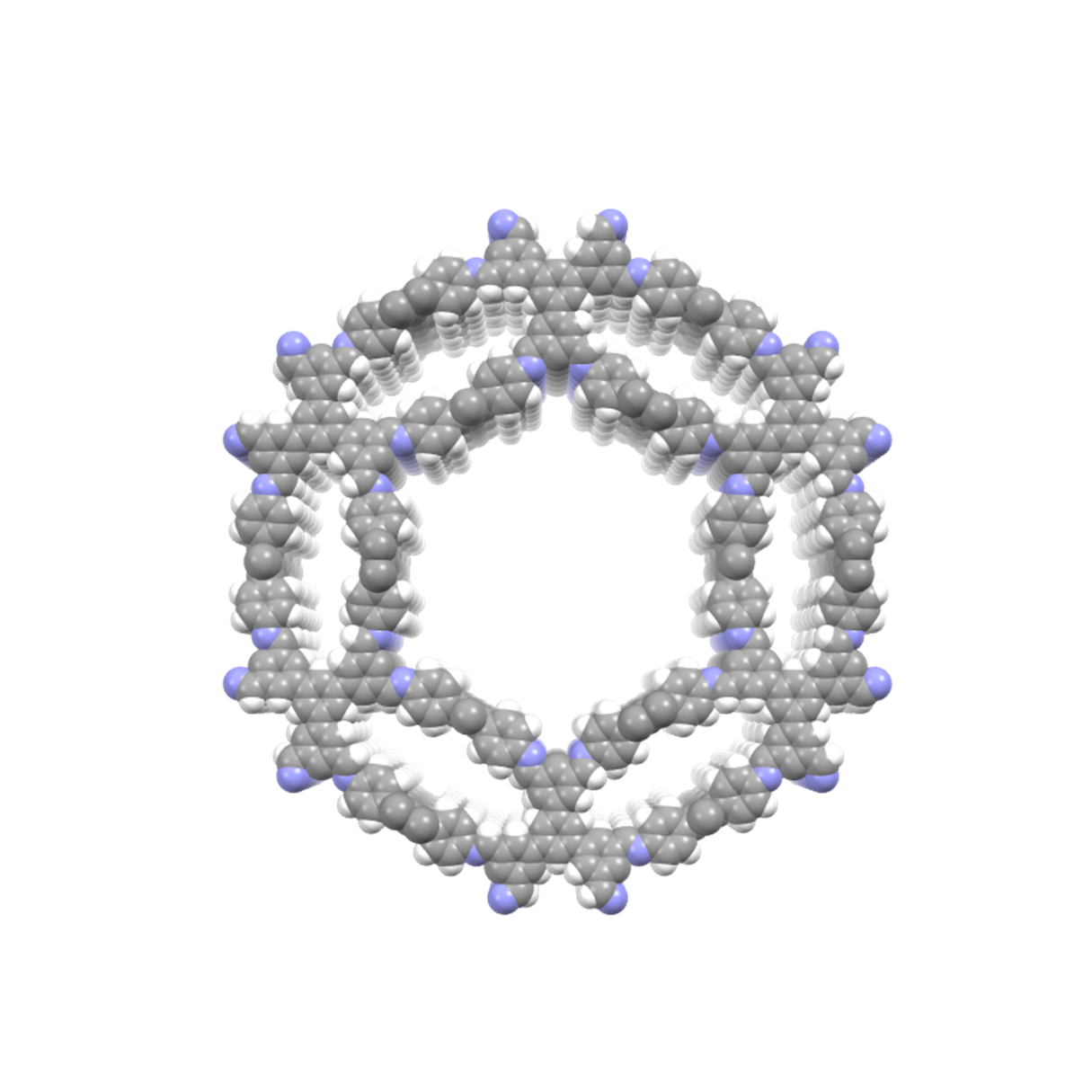


**Figure S19.** Space fill model of **M-COF** based on the **zyg** net.


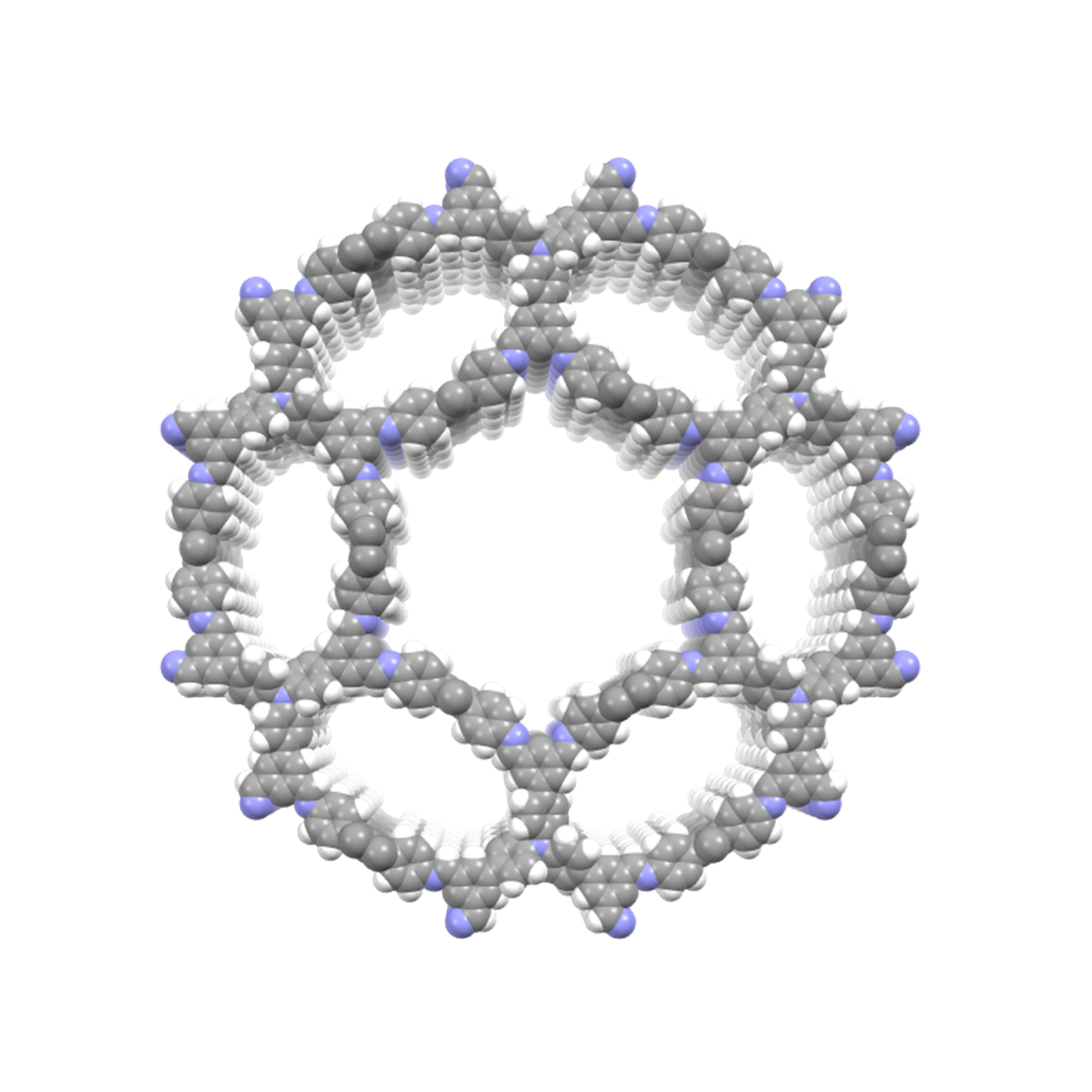


**Figure S20.** Space fill model of **N-COF** based on the **zyg** net.


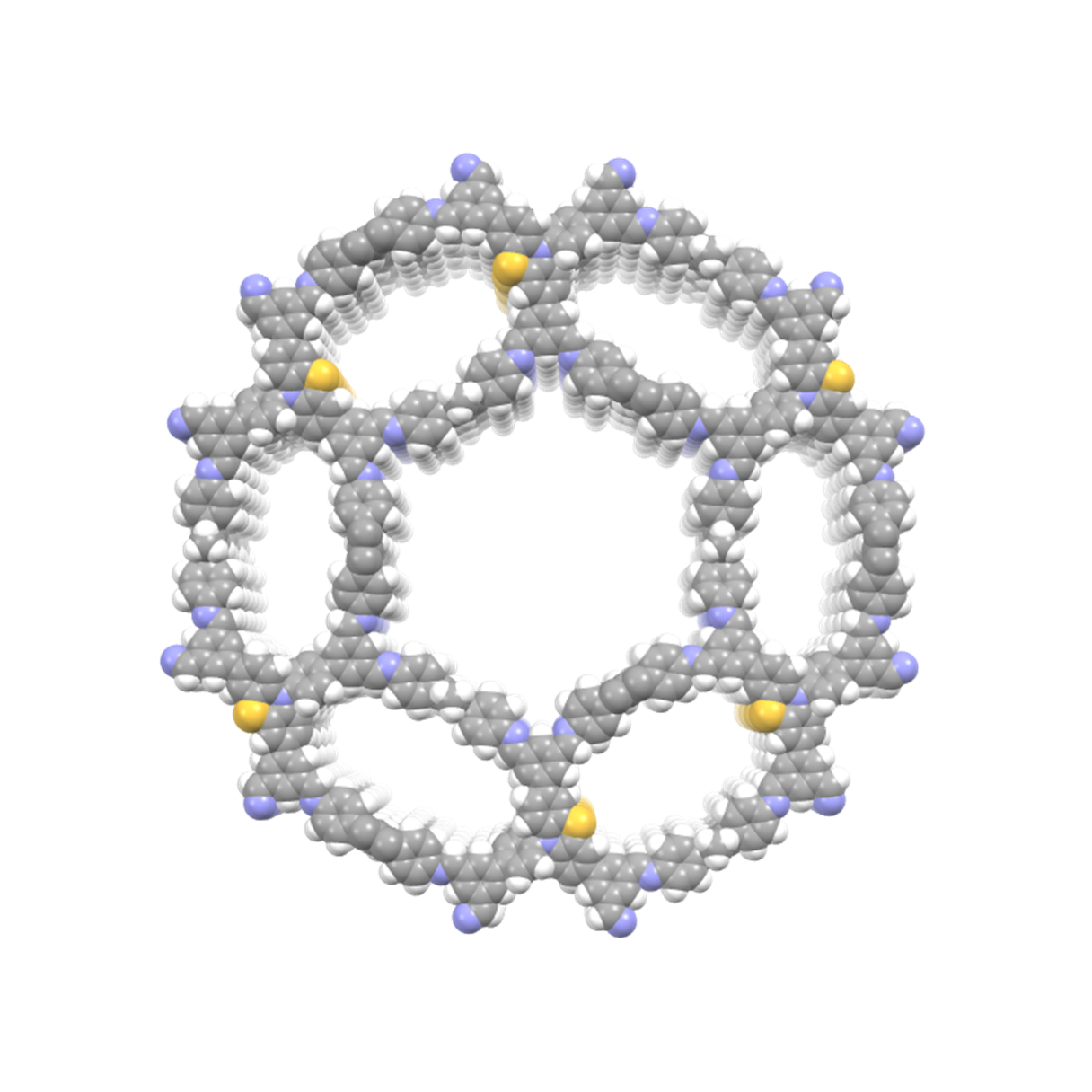


**Figure S21.** Space fill model of **S-COF** based on the **zyg** net.


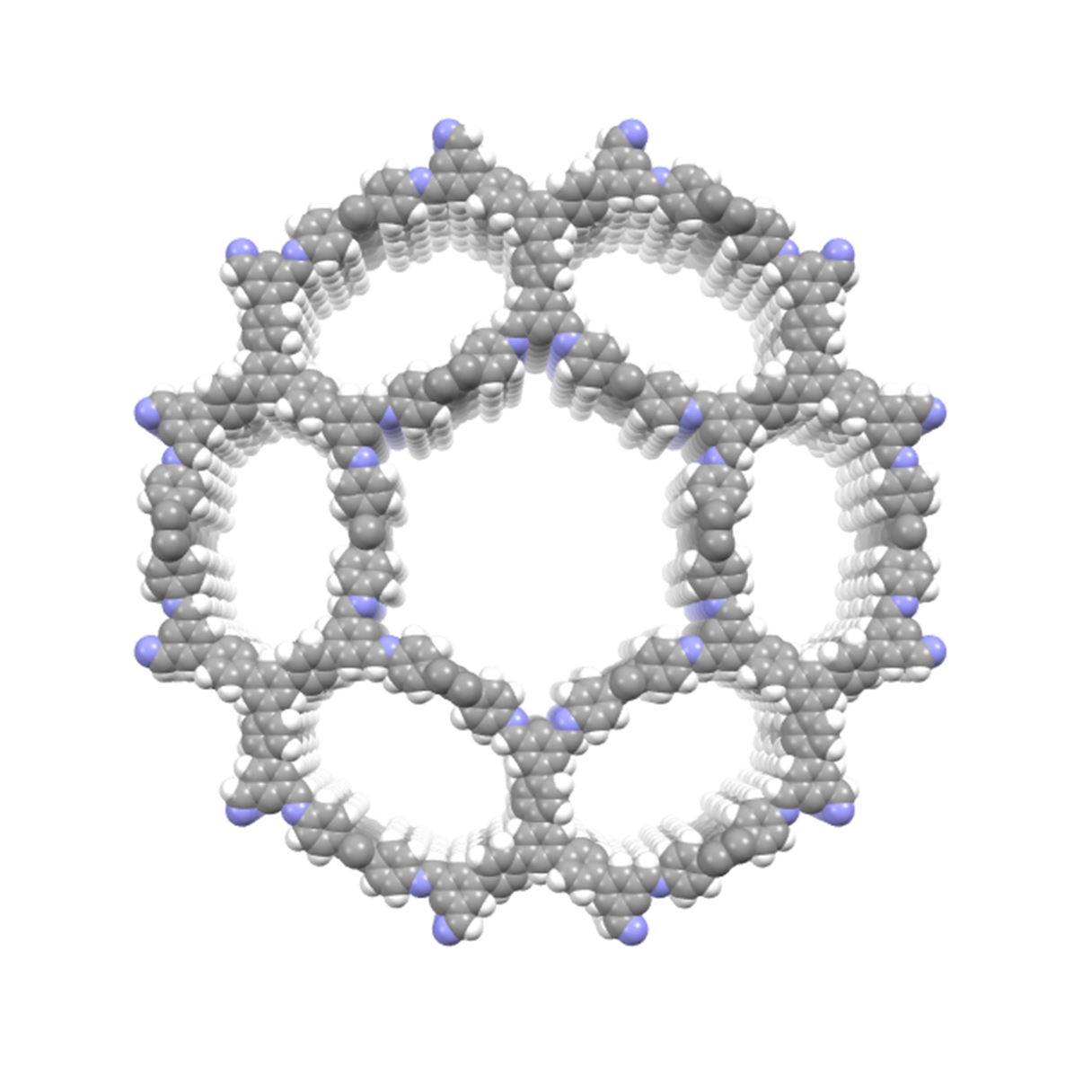


**Figure S22.** Space fill model of **T-COF** based on the **zyg** net.

**
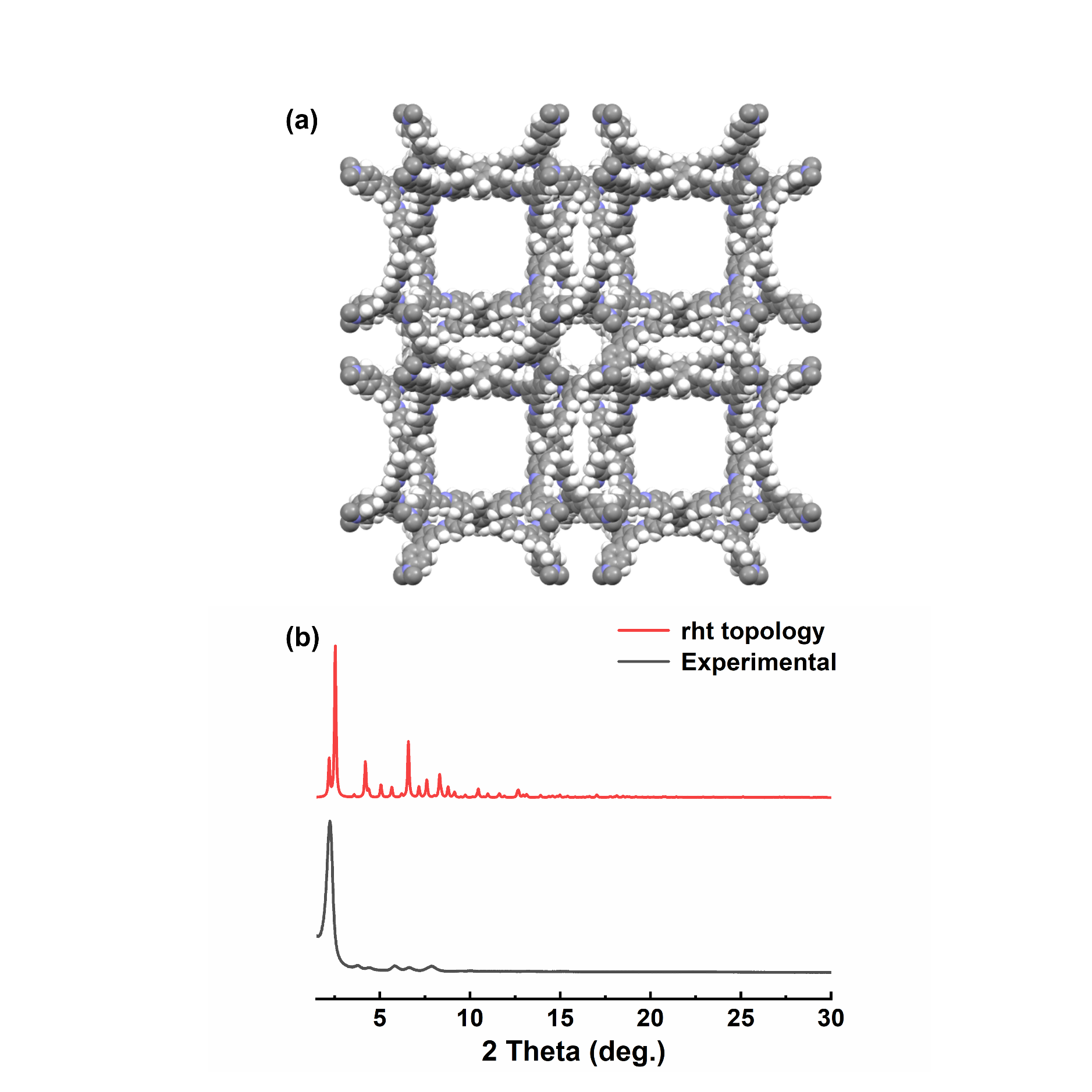
**

**Figure S23.** Space fill model (a) of **N-COF** and comparison of XRD patterns between the calculated and experimental data calculated XRD patterns (b) based on the **rht** net.


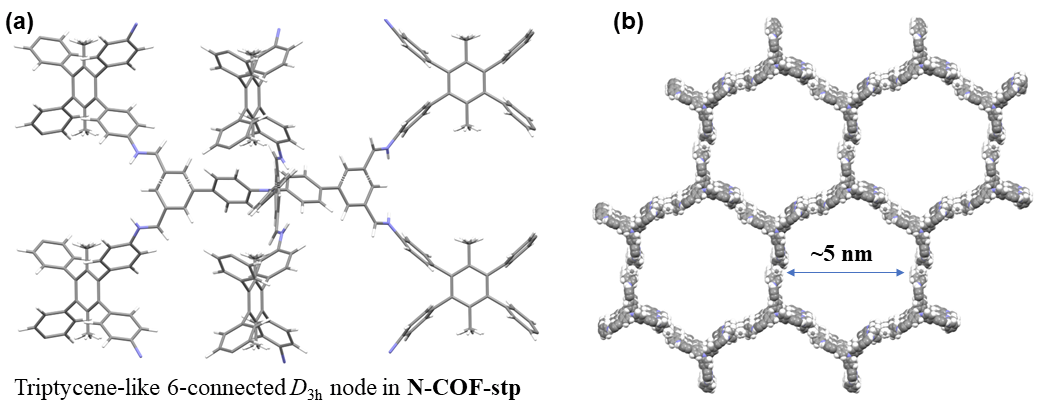


**Figure S24.** The structure of **N-COF** modeled with **stp** topology (termed as **N-COF-stp**). In the structure of **N-COF-stp**, the hexagonal node has the conformation of triptycene acting as 6-connected *D*_3h_ node (a); **N-COF-stp** has 1D channel with diameter of ~5 nm, which is significantly larger than the pore dimension (3.4 nm) obtained from the pore size distribution analysis from the N_2_ adsorption isotherm at 77 K.

## S1.10. Transmission electron microscopy and MicroED


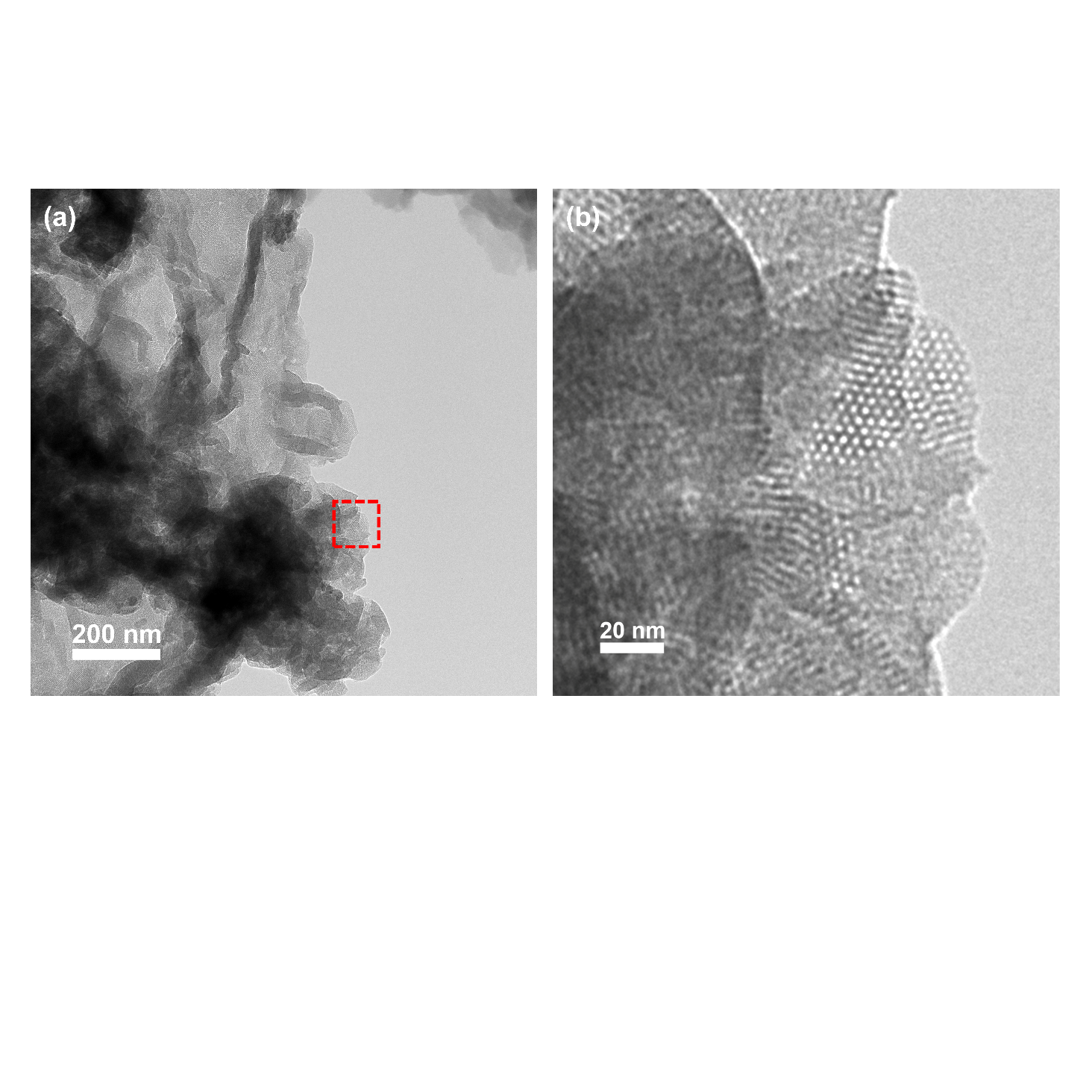


**Figure S25.** (a) TEM image of **M-COF**. (b) Magnification of the area selected in (a) as marked in red box.


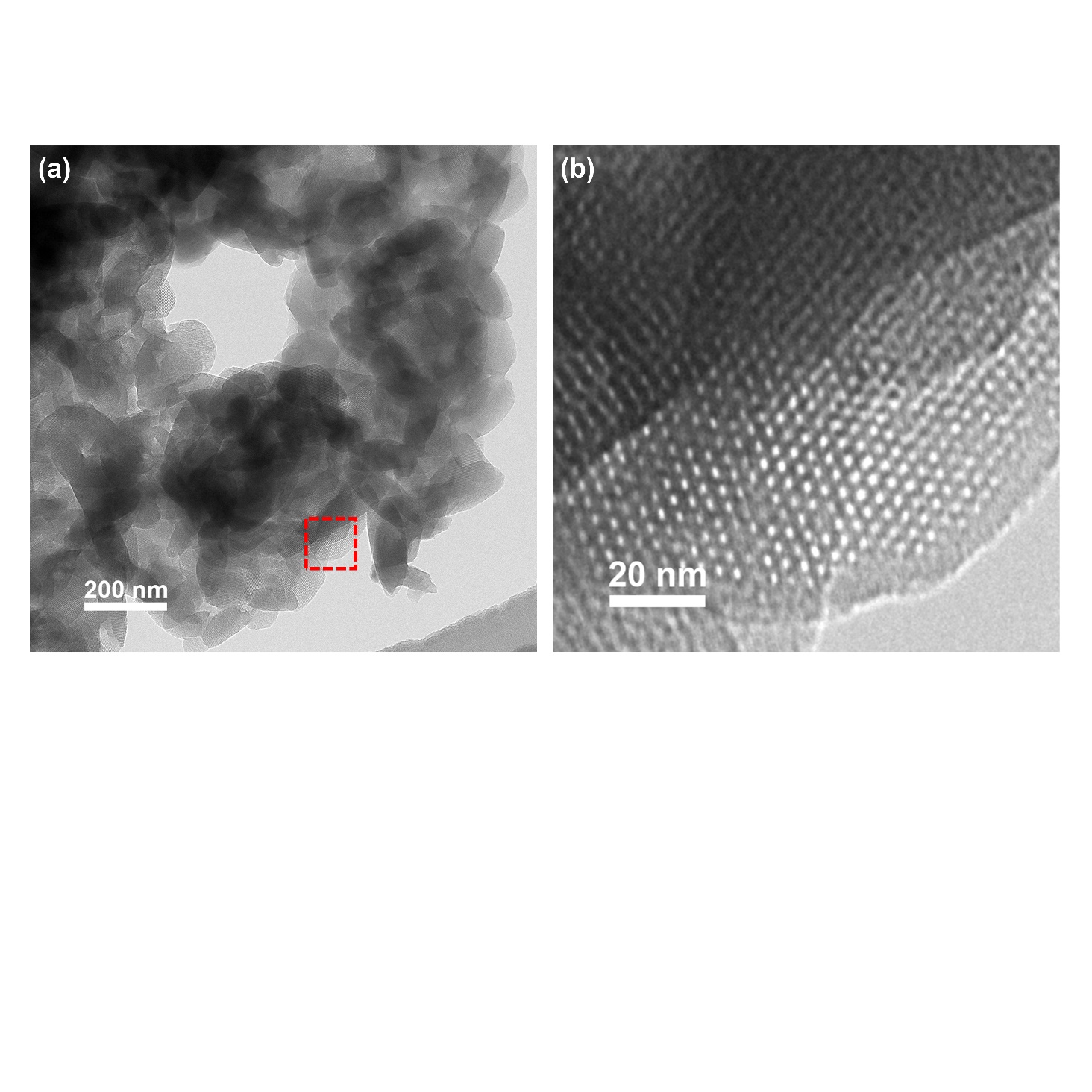


**Figure S26.** (a) TEM image of **N-COF**. (b) Magnification of the area selected in (a) as marked in red box.

**
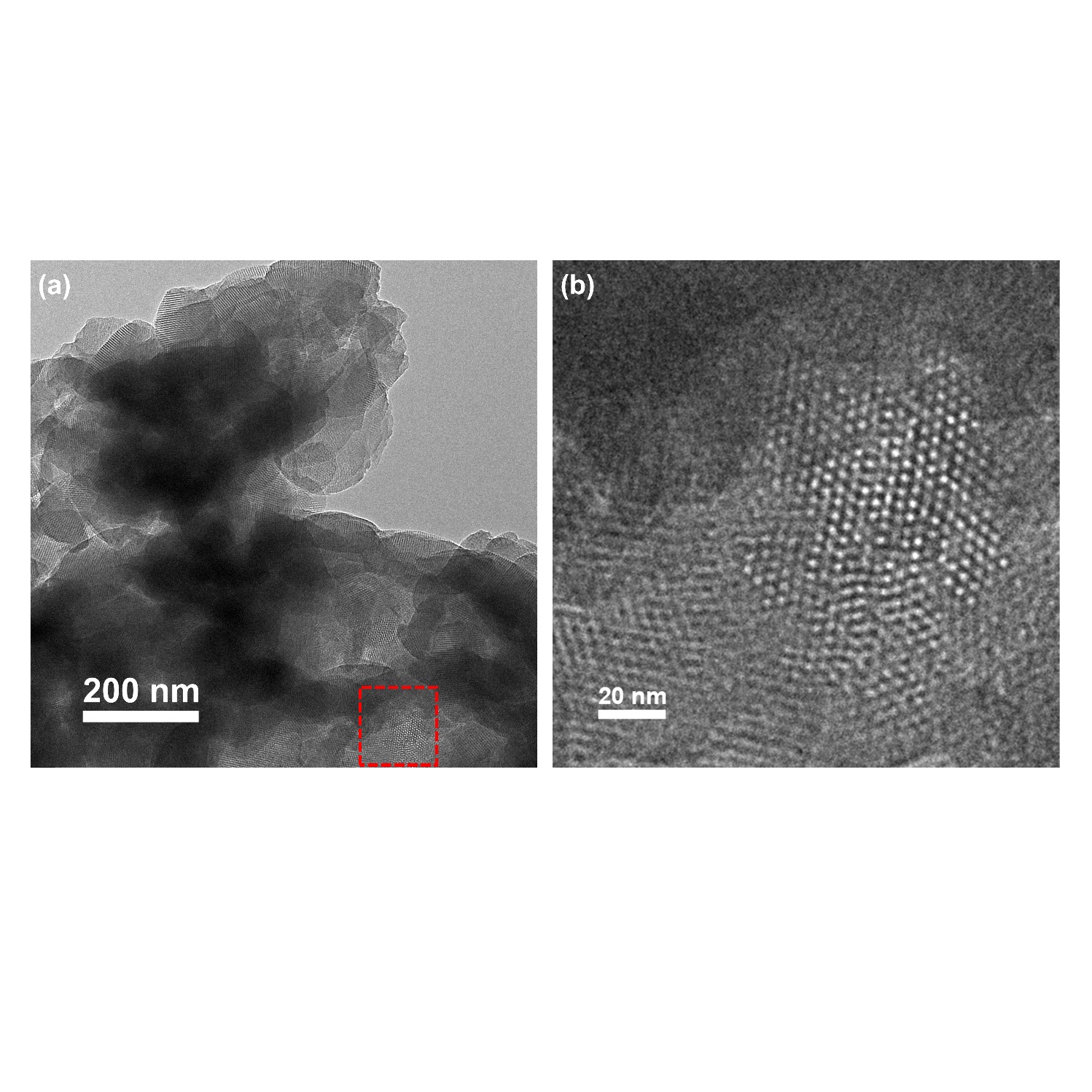
**

**Figure S27.** (a) TEM image of **S-COF**. (b) Magnification of the area selected in (a) as marked in red box.

**Micro electron diffraction (MicroED).** MicroED experiments for the COF samples were conducted. The electron diffraction data collection was performed on a JEM-2100 Plus TEM with acceleration voltage of 200 kV, λ = 0.0025079 nm, equipped with MerelinEM high-speed camera. The COF samples were loaded on a QuantiFoil (R1.2/1.3) with a Fischione 2550 cryo-transfer (the sample temperature was maintained at around 100 K).

**
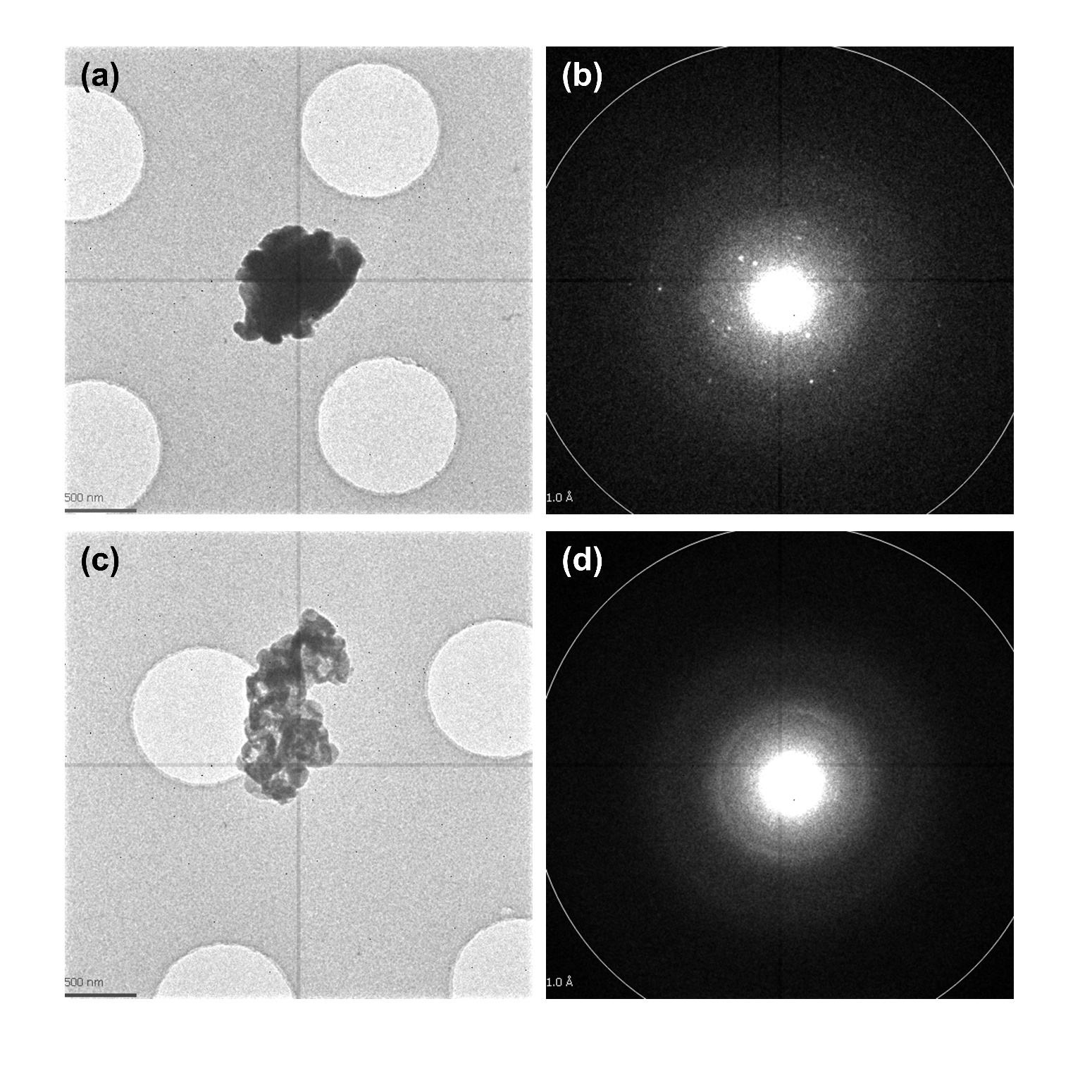
**

**Figure S28.** (a) TEM image of **N-COF**. (b) The electron diffraction pattern of (a). (c) TEM image of **S-COF**. (d) The electron diffraction pattern of (c). (Image description: the sample crystals are agglomerated and poorly diffractive. No crystals with good diffraction points were found.)

## S1.11. Gas sorption data





**Figure S29.** BET surface area plot for **M-COF**.





**Figure S30.** BET surface area plot for **N-COF**.





**Figure S31.** BET surface area plot for **S-COF**.





**Figure S32.** BET surface area plot for **T-COF**.

## S1.12. Electron paramagnetic resonance


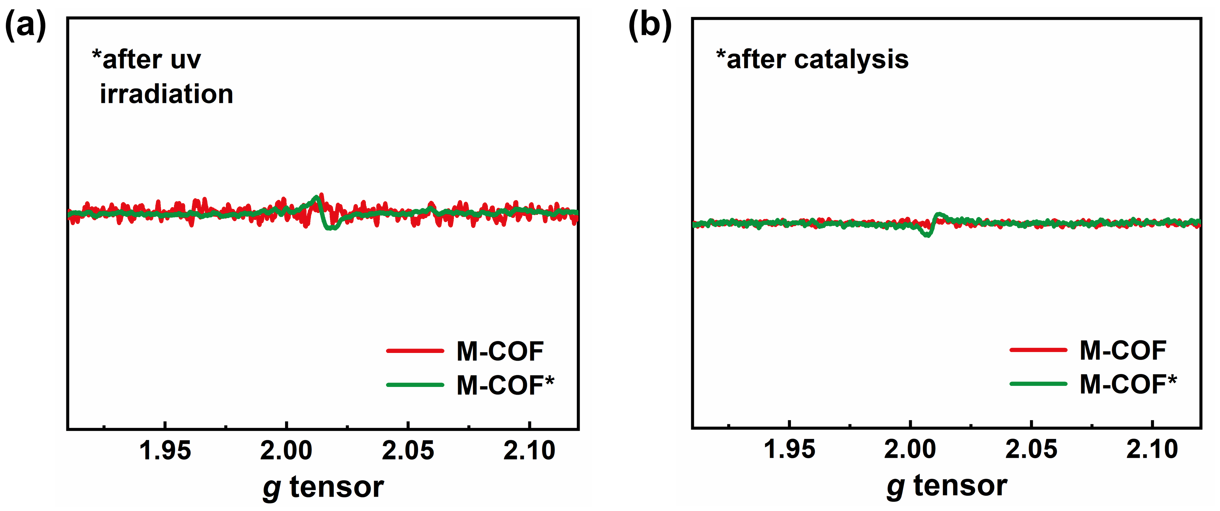


**Figure S33.** EPR spectra for as-prepared sample of **M-COF** and its irradiated sample in solid state (a) and in catalytic reaction condition (b).


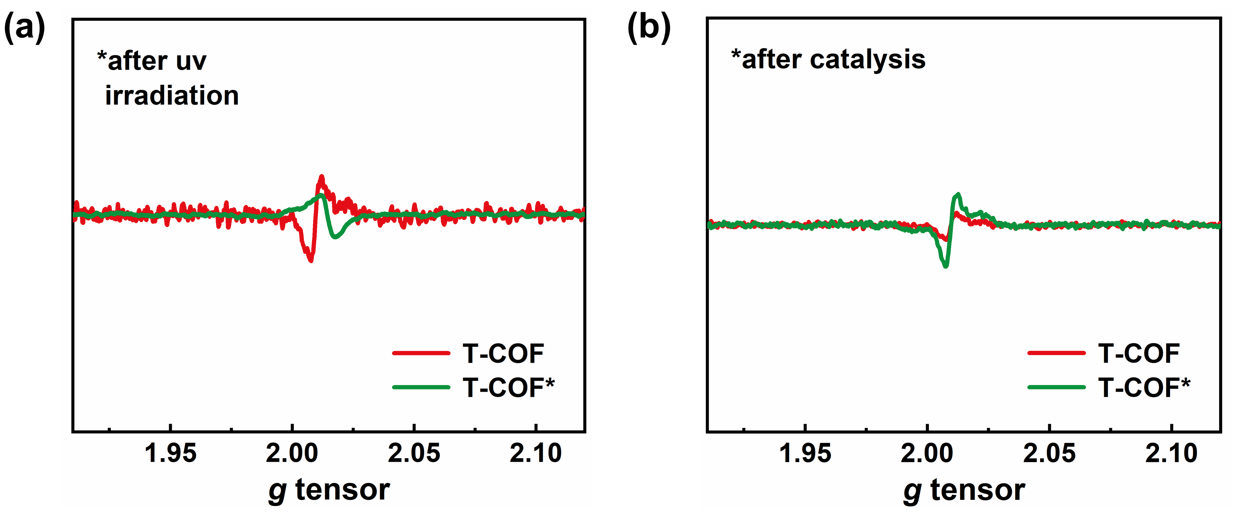


**Figure S34.** EPR spectra for as-prepared sample of **T-COF** and its irradiated sample in solid state (a) and in catalytic reaction condition (b).


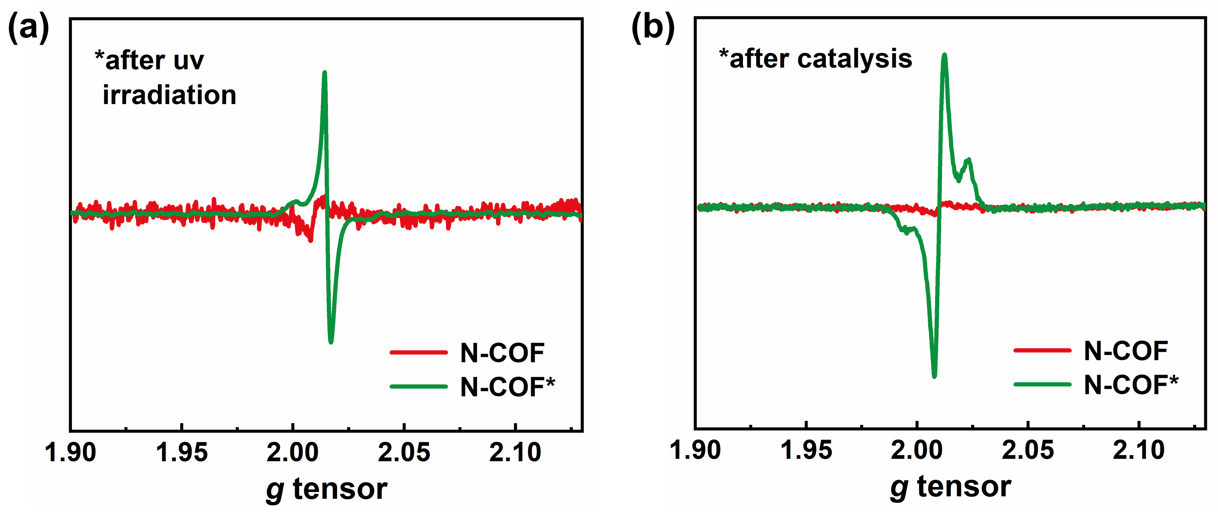


**Figure S35.** EPR spectra for as-prepared sample of **N-COF** and its irradiated sample in solid state (a) and in catalytic reaction condition (b).


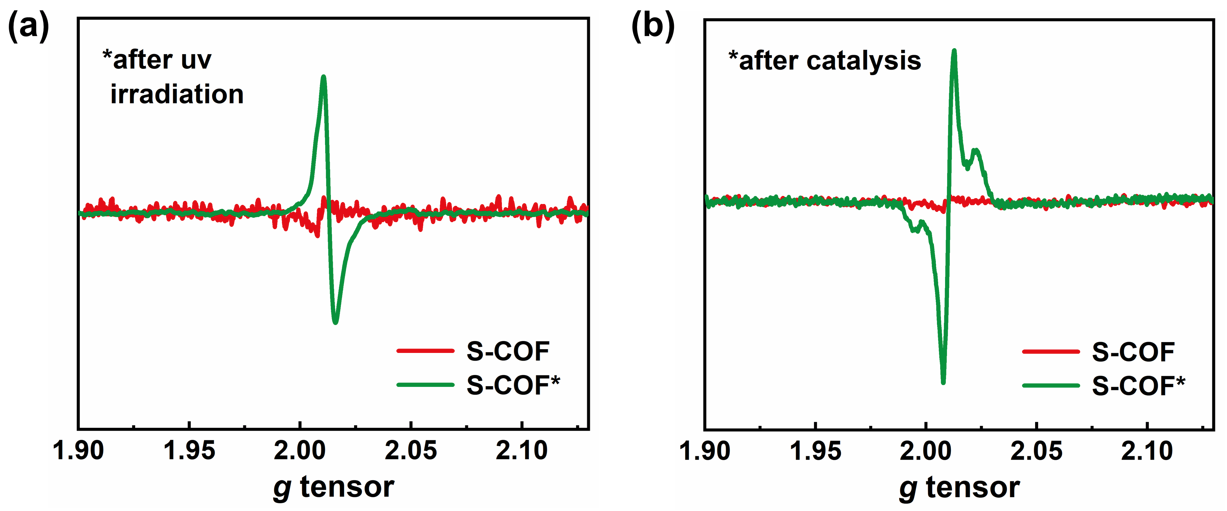


**Figure S36.** EPR spectra for as-prepared sample of **S-COF** and its irradiated sample in solid state (a) and in catalytic reaction condition (b).

# S2. Photocatalytic Experiments and Photoelectrochemical Measurements

## S2.1. Photocatalytic experiments and characterizations

**Photoelectrochemical measurements.** All electrochemical measurements (photocurrent, Mott Schottky and EIS) were performed in 0.5 M sodium sulfate solution (pH = 6.8) through the traditional three electrode system in the CHI 660E electrochemical workstation. The working electrode is ITO glass plate coated with the catalyst slurry, and the counter electrode is platinum foil. Saturated Ag/AgCl was used as reference electrode. Mott Schottky plots were measured at alternating current (AC) frequencies of 500 Hz, 1000 Hz and 1500 Hz.

**Preparation of working electrode.** 2 mg of catalyst, 990 μL of ethanol and 10 μL of Nafion D-520 were mixed and sonicated for 30 minutes. Then, 200 μL of slurry was deposited evenly on the ITO glass plate (1 × 2 cm^2^) and left in the air to dry.

**Photocatalytic reactions.** In a typical experiment, the photosynthesis of H_2_O_2_ was carried out in 25 mL glass tube containing 5 mg COF and 10 mL of deionized water at 25 ºC under O_2_ atmosphere. Then, the system was irradiated with a xenon lamp source (PLS-SXE300+, Beijing Perfect Light) or a monochromatic light at 380 nm from a multi-channel photocatalytic reaction system (PCX-50C, Beijing Perfect Light). Samples were taken after every 30 minutes and the H_2_O_2_ content of the solution was measured after filtering the catalyst.

**Isotope calibration.** The ^18^O_2_ isotope was measured on the mass spectrometer (Agilent GCMS-8890-5977B). 10 mL of ^18^O_2_, 3 mL of H_2_O, and 5 mg of COF sample were added to a reactor (10 mL). After that, the mixture was mixed well and irradiated with a 300 W xenon lamp (λ > 420 nm) for 12 h. After injecting N_2_ to replace O_2_ in a new reactor (5 mL) with above photocatalytic H_2_O_2_ solution (2 mL), an appropriate amount of MnO_2_ was added to generate O_2_. Finally, the O_2_ was detected by mass spectrometry.

**AQY measurement.** The apparent quantum yield (AQY) was determined under monochromatic LED light irradiation at a certain wavelength (λ = 380nm, 420 nm, 520 nm or 620 nm), and the light intensity was measured by a CEL-NP2000-2(10)A with a photodiode sensor.

The AQY was calculated using the following equation:


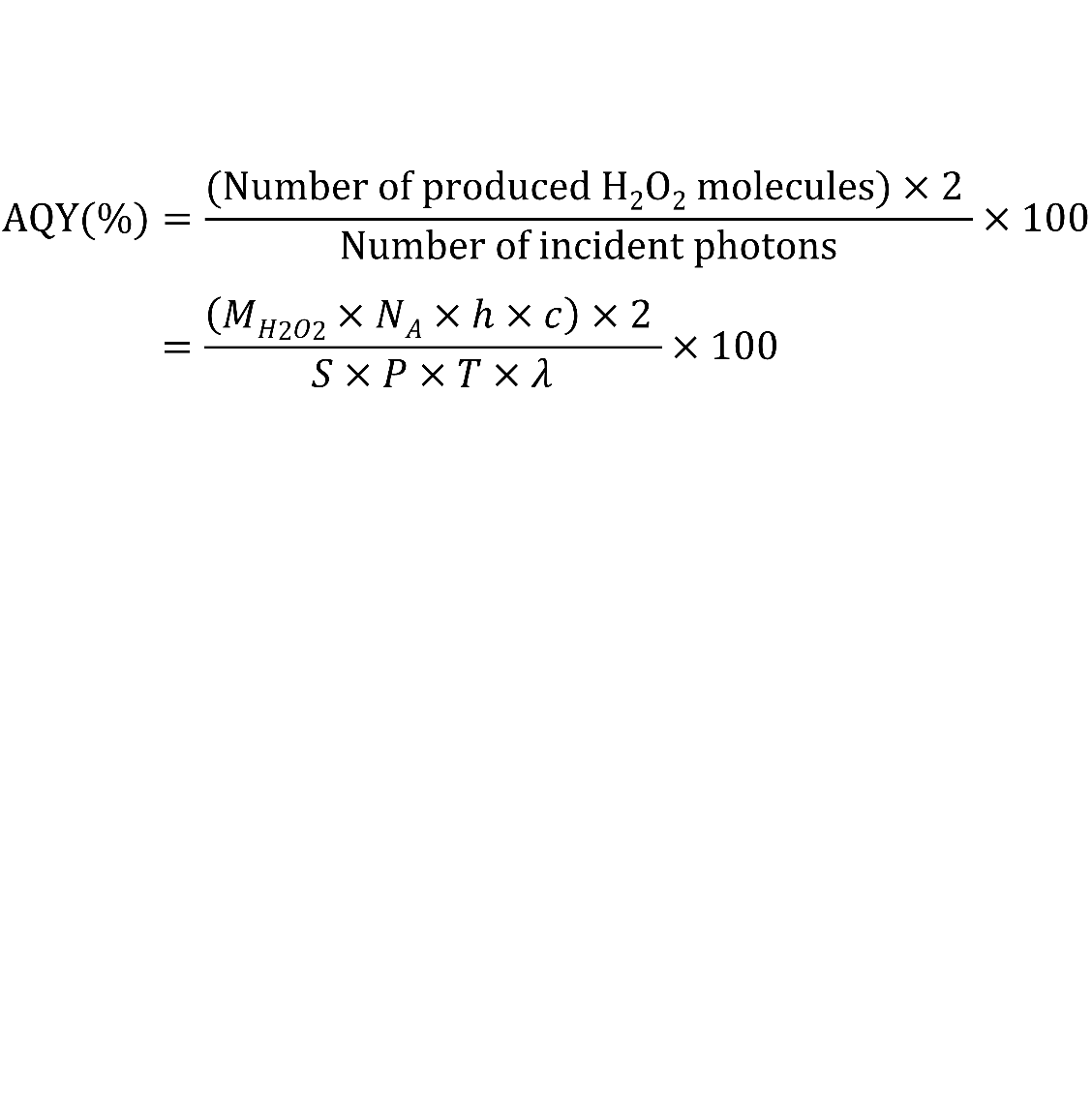


*M* = yield of H_2_O_2_ (mol); *N_A_* = 6.02 × 10^23^ mol^-1^; *h* = 6.626 × 10^-34^ J s; *c* = 3 × 10^8^ m/s; *S* = irradiation area (cm^2^) = 7.07 cm^2^; *P* = the intensity of irradiation light (W / cm^2^) = 192 mW / cm^2^ (380 nm), 83 mW / cm^2^ (420 nm), 81 mW / cm^2^ (520 nm), 80 mW / cm^2^ (620 nm); *T* = the photoreaction time (s) = 3600 s; *λ* = the wavelength of the monochromatic light (10^-9^ m).

Calculation after tests:

**N-COF*:** AQY (380 nm) = 0.13%; AQY (420 nm) = 0.10%; AQY (520 nm) = 0.02%; AQY (620 nm) = 0.006%.

**S-COF*:** AQY (380 nm) = 0.41%; AQY (420 nm) = 0.19%; AQY (520 nm) = 0.02%; AQY (620 nm) = 0.005%.

## S2.2. Mott-Schottky measurements





**Figure S37.** Mott-Schottky plots of **N-COF**.





**Figure S38.** Mott-Schottky plots of **N-COF***.





**Figure S39.** Mott-Schottky plots of **S-COF**.





**Figure S40.** Mott-Schottky plots of **S-COF***.

## S2.3. Transient photocurrent response

**

**

**Figure S41.** Transient photocurrent responses of **N-COF** and **N-COF***.





**Figure S42.** Transient photocurrent responses of **S-COF** and **S-COF***.

## S2.4. Stability tests

The post characterization of catalysts was conducted using the following sample processing method: after the photocatalytic reaction, the sample was scraped from the reactor and collected, then dried in a 120 °C vacuum oven for 12 hours to perform structural characterizations.

**

**

**Figure S43.** PXRD patterns of **N-COF** before and after photocatalytic reactions.

**

**

**Figure S44.** PXRD patterns of **S-COF** before and after photocatalytic reactions.

**

**

**Figure S45.** FT-IR patterns of **N-COF** before and after photocatalytic reactions.

**

**

**Figure S46.** FT-IR patterns of **S-COF** before and after photocatalytic reactions.

## S2.5. H_2_O_2_ detection methods

**Ce(SO_4_)_2_ titration.** The purified sample (1 mL) was diluted for an appropriate number of times and transferred into an EP plastic tube (5 mL), then 1.5 ml of the solution was taken and mixed with 1.5 mL of solutions containing cerium sulfate (Ce(SO_4_)_2_, 1 mmol/L) and sulfuric acid solution (H_2_SO_4_, 0.2 mol/L). H_2_O_2_ reduces a yellow Ce^4+^ solution to colourless (Ce^3+^). The absorbance of Ce^4+^ at 318 nm was calibrated against the concentration of H_2_O_2_ using a UV-Vis spectrophotometer (Agilent Cary 5000) after using a standard solution of H_2_O_2_ (Figure S47). Based on the linear relationship, the concentration of H_2_O_2_ in the sample can be obtained.

**HRP/TMB colorimetric method.** The purified sample (1 mL) was diluted for an appropriate number of times and transferred into an EP plastic tube (5 mL), then 0.15 ml of the solution was taken and mixed with 1.35 ml of water, 1.5 mL of a solution containing acetate buffer (0.1 M, pH 3.5), 3,3’,5,5’-tetramethylbenzidine (TMB, 100 µmol/L) and peroxidase from horseradish (≥300 units/mg solid, HRP, 50 µg/mL). The reaction of colourless TMB with H_2_O_2_ catalyzed by HRP produced a blue TMB-Ox product. The absorbance of the TMB-Ox at 652 nm against the concentration of H_2_O_2_ was calibrated with a UV-Vis spectrophotometer (Agilent Cary 5000) after using a standard solution of H_2_O_2_ (Figure S48). Based on the linear relationship, the concentration of H_2_O_2_ in the sample can be obtained.


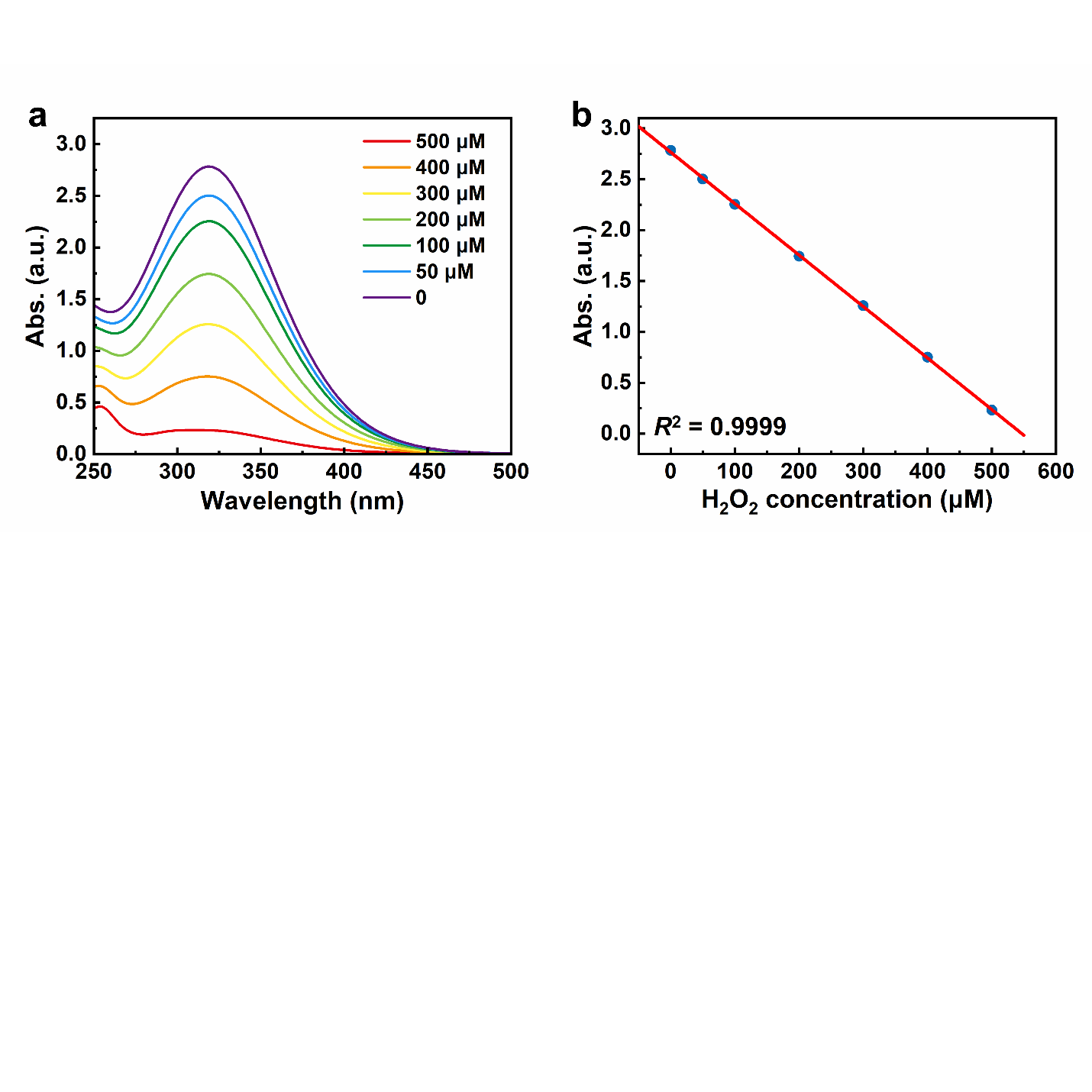


**Figure S47.** The standard curve of H_2_O_2_ concentration-absorbance by Ce(SO_4_)_2_ titration. (a) UV−vis absorption spectra of Ce^4+^ solutions with different concentrations. (b) Corresponding calibration curve at the absorption peak of 318 nm.


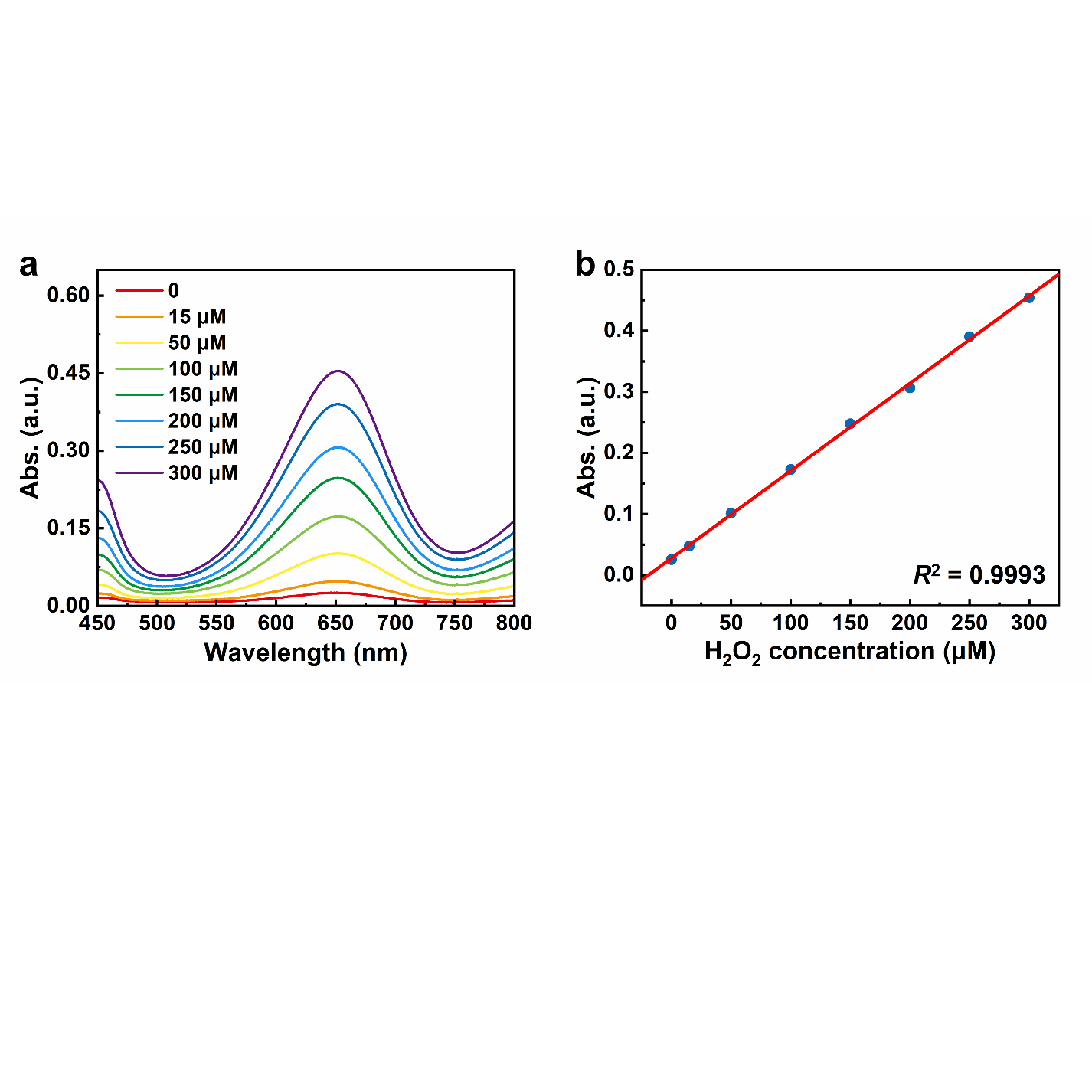


**Figure S48.** The standard curve of H_2_O_2_ concentration-absorbance by HRP/TMB colorimetric method. (a) UV−vis absorption spectra of TMB-Ox solutions with different concentrations. (b) Corresponding calibration curve at the absorption peak of 652 nm.

## S2.6. Isotope calibration





**Figure S49.** ^18^O_2_ isotope experiment of **N-COF*** to explore the source of H_2_O_2_.

## S2.7. *In-situ* electron paramagnetic resonance





**Figure S50.** EPR spectrum of the reaction solution under the dark and visible light illumination for **N-COF*** in the presence of DMPO as the spin-trapping reagents.

## S2.8. DFT calculation

All gas-phase geometry optimizations for the ground states were carried out using the Gaussian 16^3^ program at (U)B3LYP-D3(BJ)/def2-SVP^4^ level. Frequency calculations were evaluated at the same theoretical level to obtain the energies of thermodynamic corrections at 298.15 K and 1 atm. The translations entropy correction was refined by employing the method developed by Whitesides^5^ to explain the change from the gas phase in the standard state to an aqueous solution. In order to obtain the electron energy with high accuracy, single-point calculations were performed at the level of SMD^6^(water)/(U)B3LYP-D3(BJ)/def2-TZVP.^7^


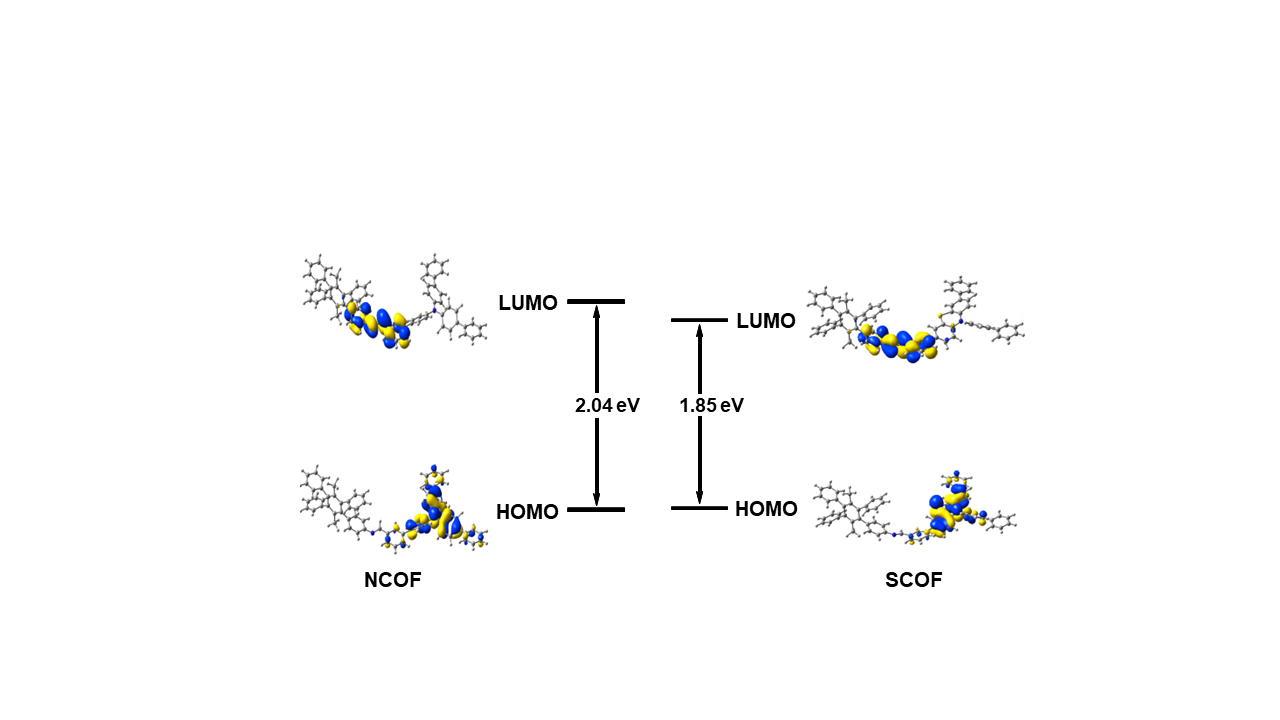


**Figure S51.** Energy band diagrams for **N-COF** and **S-COF**.

**Table S1.** Summary of H_2_O_2_ photosynthesis without sacrificial agents by COF-based photocatalysts.

| **Material** | **Light** | **Conditions** | **[Cat.] (g L^-1^)** | **Rate (umol h^-1^g^-1^)** | **Ref.** |
| --- | --- | --- | --- | --- | --- |
| **S-COF*** | **380nm** | **H_2_O, O_2_** | **0.333** | **3324** | **This work** |
| **S-COF*** | **xenon lamp** | **H_2_O, O_2_** | **0.5** | **1160** | **This work** |
| **N-COF*** | **380nm** | **H_2_O, O_2_** | **0.333** | **1140** | **This work** |
| **N-COF*** | **xenon lamp** | **H_2_O, O_2_** | **0.5** | **710** | **This work** |
| COF-TTA-TTTA | >420 nm | H_2_O, O_2_ | 0.3 | 2406 | ref. 8 |
| COF-TfpBpy | 420-700 nm | H_2_O, air | 1.5 | 694.7 | ref. 9 |
| sonoCOF-F2 | >420 nm | H_2_O, O_2_ | 0.6 | 2736 | ref. 10 |
| Bpt-CTF | 350-780 nm | H_2_O, O_2_ | 0.2 | 3268.1 | ref. 11 |
| 4PE-N-S COF | 420-700 nm | H_2_O, O_2_ | 0.5 | 1574 | ref. 12 |
| DMCR-1NH | >420 nm | H_2_O, air | 0.455 | 2264.5 | ref. 13 |
| PMCR-1 | 400-700nm | H_2_O, O_2_ | 0.455 | 1445 | ref. 14 |
| Bpy-TAPT | >420 nm | H_2_O, O_2_ | 0.167 | 4038 | ref. 15 |
| CHF-DPDA | >420 nm | H_2_O, O_2_ | 2 | 1725 | ref. 16 |
| HEP-TAPT-COF | >420 nm | H_2_O, O_2_ | 0.5 | 1750 | ref. 17 |
| TpDz | >420 nm | H_2_O, O_2_ | 0.167 | 7327 | ref. 18 |
| N_0_-COF | 495 nm LED | H_2_O, O_2_ | 0.5 | 1570 | ref. 19 |
| TPB-DMTP-COF | >420 nm | H_2_O, O_2_ | 0.2 | 2882 | ref. 20 |
| TTF-BT-COF | >420 nm | H_2_O, O_2_ | 0.5 | 2760 | ref. 21 |
| TaptBtt | >420 nm | H_2_O, air | 1.2 | 1407 | ref. 22 |

# S3. Crystallographic Information

**Table S2.** Fractional atomic coordinates for **M-COF** modelled based on the **zyg** net.

| **M-COF** | | | |
| --- | --- | --- | --- |
| **Space group** | *P*6_3_/*mcm* | | |
| **Calculated unit cell parameters** | *a* = *b* = 43.1693 Å, *c* = 9.1362 Å  *α* = *β* = 90°, *γ* = 120° | | |
| **Atom** | **x** | **y** | **z** |
| N1 | 0.17913 | 0.7003 | 0.51823 |
| C1 | 0.29606 | 0.64803 | 0.5 |
| H1 | 0.26746 | 0.63373 | 0.5 |
| C2 | 0.31427 | 0.68573 | 0.5 |
| C3 | 0.29402 | 0.70598 | 0.5 |
| C4 | 0.25762 | 0.68927 | 0.54168 |
| H4 | 0.24352 | 0.662 | 0.58013 |
| C5 | 0.23824 | 0.70733 | 0.5332 |
| C6 | 0.25612 | 0.74388 | 0.5 |
| H6 | 0.24163 | 0.75837 | 0.5 |
| C7 | 0.19924 | 0.68722 | 0.55755 |
| H7 | 0.18764 | 0.66092 | 0.60649 |
| C8 | 0.14086 | 0.68209 | 0.53228 |
| C9 | 0.12395 | 0.70253 | 0.54179 |
| H9 | 0.13954 | 0.73138 | 0.53306 |
| C10 | 0.08744 | 0.68618 | 0.57385 |
| H10 | 0.07539 | 0.70262 | 0.59245 |
| C11 | 0.06745 | 0.64909 | 0.59538 |
| C12 | 0.08352 | 0.62823 | 0.57207 |
| H12 | 0.06835 | 0.59954 | 0.58834 |
| C13 | 0.11998 | 0.64454 | 0.54035 |
| H13 | 0.13178 | 0.62777 | 0.52662 |
| C14 | 0.03257 | 0.63309 | 0.67246 |
| C15 | 0 | 0.61658 | 0.59437 |
| C16 | 0 | 0.61671 | 0.42909 |
| H16 | 0.02366 | 0.61634 | 0.38417 |
| H17 | 0 | 0.64102 | 0.38961 |

**Table S3.** Fractional atomic coordinates for **M-COF** derived from Rietveld refinement.

| **M-COF** | | | |
| --- | --- | --- | --- |
| **Space group** | *P*6_3_/*mcm* | | |
| **Calculated unit cell parameters** | *a* = *b* = 43.1873 Å, *c* = 8.9426 Å  *α* = *β* = 90°, *γ* = 120° | | |
| **Atom** | **x** | **y** | **z** |
| N1 | 0.178933 | 0.700534 | 0.517968 |
| C1 | 0.295814 | 0.648294 | 0.499232 |
| H1 | 0.267227 | 0.633998 | 0.499228 |
| C2 | 0.314015 | 0.685975 | 0.499267 |
| C3 | 0.293773 | 0.706216 | 0.499299 |
| C4 | 0.257387 | 0.689508 | 0.541877 |
| H4 | 0.243293 | 0.662248 | 0.581135 |
| C5 | 0.238016 | 0.70756 | 0.533239 |
| C6 | 0.255887 | 0.744096 | 0.499359 |
| H6 | 0.241402 | 0.758579 | 0.499381 |
| C7 | 0.199034 | 0.687458 | 0.558112 |
| H7 | 0.187442 | 0.661169 | 0.608081 |
| C8 | 0.14068 | 0.68233 | 0.53231 |
| C9 | 0.123777 | 0.70276 | 0.542057 |
| H9 | 0.13936 | 0.731599 | 0.53316 |
| C10 | 0.087283 | 0.686414 | 0.574806 |
| H10 | 0.075236 | 0.702844 | 0.593827 |
| C11 | 0.067303 | 0.649341 | 0.596766 |
| C12 | 0.083365 | 0.628491 | 0.57292 |
| H12 | 0.068203 | 0.599813 | 0.589521 |
| C13 | 0.11981 | 0.644798 | 0.540517 |
| H13 | 0.131606 | 0.628036 | 0.526471 |
| C14 | 0.032437 | 0.633342 | 0.675507 |
| C15 | -0.00012 | 0.616845 | 0.595732 |
| C16 | -0.00012 | 0.616982 | 0.42688 |
| H16 | 0.023534 | 0.616616 | 0.38098 |
| H17 | -0.00012 | 0.641285 | 0.386574 |

**Table S4.** Fractional atomic coordinates for **N-COF** modelled based on the **zyg** net.

| **N-COF** | | | |
| --- | --- | --- | --- |
| **Space group** | *P*6_3_/*mcm* | | |
| **Calculated unit cell** | *a* = *b* = 47.7677 Å, *c* = 9.3739 Å  *α* = *β* = 90°, *γ* = 120° | | |
| **Atom** | **x** | **y** | **z** |
| N1 | 0.33333 | 0.66667 | 0.5 |
| N2 | 0.16026 | 0.73262 | 0.50831 |
| C1 | 0.31575 | 0.68425 | 0.5 |
| C2 | 0.28592 | 0.67187 | 0.42921 |
| C3 | 0.26877 | 0.68846 | 0.43128 |
| C4 | 0.28151 | 0.71849 | 0.5 |
| C5 | 0.26356 | 0.73644 | 0.5 |
| C6 | 0.22976 | 0.71986 | 0.51471 |
| C7 | 0.21262 | 0.73659 | 0.51279 |
| C8 | 0.2296 | 0.7704 | 0.5 |
| C9 | 0.17713 | 0.7184 | 0.52715 |
| C10 | 0.12588 | 0.71807 | 0.52533 |
| C11 | 0.1051 | 0.68436 | 0.53374 |
| C12 | 0.07268 | 0.67177 | 0.57069 |
| C13 | 0.0606 | 0.69257 | 0.59872 |
| C14 | 0.08051 | 0.7259 | 0.5784 |
| C15 | 0.11295 | 0.73852 | 0.54148 |
| C16 | 0.02938 | 0.67997 | 0.67439 |
| C17 | 0 | 0.66595 | 0.59814 |
| C18 | 0 | 0.66565 | 0.43666 |
| H2 | 0.27563 | 0.64909 | 0.37311 |
| H3 | 0.24574 | 0.67799 | 0.37694 |
| H6 | 0.21668 | 0.69385 | 0.52855 |
| H8 | 0.21651 | 0.78349 | 0.5 |
| H9 | 0.16571 | 0.69328 | 0.55833 |
| H11 | 0.11377 | 0.66764 | 0.51635 |
| H12 | 0.05747 | 0.64595 | 0.586 |
| H14 | 0.07147 | 0.74222 | 0.60038 |
| H15 | 0.12853 | 0.76446 | 0.53352 |
| H18 | 0.02143 | 0.68737 | 0.39287 |
| H19 | 0 | 0.64367 | 0.39825 |

**Table S5.** Fractional atomic coordinates for **N-COF** derived from Rietveld refinement.

| **N-COF** | | | |
| --- | --- | --- | --- |
| **Space group** | *P*6_3_/*mcm* | | |
| **Calculated unit cell** | *a* = *b* = 46.1576 Å, *c* = 9.1132 Å  *α* = *β* = 90°, *γ* = 120° | | |
| **Atom** | **x** | **y** | **z** |
| N1 | 0.33333 | 0.66667 | 0.5 |
| N2 | 0.15836 | 0.73912 | 0.44285 |
| C1 | 0.31518 | 0.68482 | 0.5 |
| C2 | 0.28407 | 0.6717 | 0.42945 |
| H2 | 0.27305 | 0.64676 | 0.36917 |
| C3 | 0.26635 | 0.68875 | 0.43192 |
| H3 | 0.24063 | 0.67725 | 0.37836 |
| C4 | 0.27986 | 0.72014 | 0.5 |
| C5 | 0.26137 | 0.73863 | 0.5 |
| C6 | 0.22726 | 0.72229 | 0.46395 |
| H6 | 0.2136 | 0.69457 | 0.43473 |
| C7 | 0.20974 | 0.73958 | 0.46336 |
| C8 | 0.22647 | 0.77353 | 0.5 |
| H8 | 0.21293 | 0.78707 | 0.5 |
| C9 | 0.17345 | 0.72179 | 0.44088 |
| H9 | 0.15936 | 0.69358 | 0.42241 |
| C10 | 0.12369 | 0.72738 | 0.46495 |
| C11 | 0.09989 | 0.69322 | 0.47513 |
| H11 | 0.10582 | 0.67376 | 0.42953 |
| C12 | 0.06913 | 0.68329 | 0.54105 |
| H12 | 0.05005 | 0.65556 | 0.5532 |
| C13 | 0.06162 | 0.70741 | 0.59246 |
| C14 | 0.08393 | 0.74137 | 0.5686 |
| H14 | 0.0767 | 0.76077 | 0.60118 |
| C15 | 0.11491 | 0.75126 | 0.505 |
| H15 | 0.13317 | 0.77897 | 0.48556 |
| C16 | 0.03025 | 0.69662 | 0.67251 |
| C17 | 0 | 0.68299 | 0.59446 |
| C18 | 0 | 0.68655 | 0.42913 |
| H18a | 0 | 0.70998 | 0.40194 |
| H18b | 0.02201 | 0.68731 | 0.37717 |
| O1 | 0.03 | 0.53045 | 0.61323 |
| O2 | 0.06769 | 0.59131 | 0.4255 |
| O3 | 0.09234 | 0.57464 | 0.63275 |

**Table S6.** Fractional atomic coordinates for **S-COF** modelled based on the **zyg** net.

| **S-COF** | | | |
| --- | --- | --- | --- |
| **Space group** | *P*6_3_/*mcm* | | |
| **Calculated unit cell** | *a* = *b* = 48.1887 Å, *c* = 9.0020 Å  *α* = *β* = 90°, *γ* = 120° | | |
| **Atom** | **x** | **y** | **z** |
| N1 | 0.33333 | 0.66667 | 0.5 |
| N2 | 0.16007 | 0.73277 | 0.51341 |
| S1 | 0.25449 | 0.62725 | 0.5 |
| C1 | 0.31557 | 0.68443 | 0.5 |
| C2 | 0.28157 | 0.66896 | 0.49103 |
| C3 | 0.26518 | 0.68613 | 0.48223 |
| C4 | 0.28078 | 0.71922 | 0.5 |
| C5 | 0.26275 | 0.73725 | 0.5 |
| C6 | 0.22977 | 0.72172 | 0.53463 |
| C7 | 0.21255 | 0.73806 | 0.52773 |
| C8 | 0.2289 | 0.7711 | 0.5 |
| C9 | 0.1775 | 0.72012 | 0.54901 |
| C10 | 0.12584 | 0.7174 | 0.52755 |
| C11 | 0.10616 | 0.68386 | 0.53587 |
| C12 | 0.07359 | 0.67028 | 0.56847 |
| C13 | 0.06031 | 0.68996 | 0.59288 |
| C14 | 0.07922 | 0.72311 | 0.57123 |
| C15 | 0.11178 | 0.73669 | 0.53795 |
| C16 | 0.02919 | 0.67673 | 0.67145 |
| C17 | 0 | 0.66281 | 0.59264 |
| C18 | 0 | 0.66285 | 0.42569 |
| H2 | 0.26761 | 0.64165 | 0.49118 |
| H3 | 0.23829 | 0.67296 | 0.45992 |
| H6 | 0.21715 | 0.69542 | 0.56869 |
| H8 | 0.21592 | 0.78408 | 0.5 |
| H9 | 0.16572 | 0.69489 | 0.5973 |
| H11 | 0.11682 | 0.66772 | 0.51599 |
| H12 | 0.05773 | 0.64313 | 0.57527 |
| H14 | 0.06804 | 0.73903 | 0.58077 |
| H15 | 0.12691 | 0.76364 | 0.51924 |
| H18 | 0.02126 | 0.68454 | 0.38154 |
| H19 | 0 | 0.6411 | 0.38525 |

**Table S7.** Fractional atomic coordinates for **S-COF** derived from Rietveld refinement.

| **S-COF** | | | |
| --- | --- | --- | --- |
| **Space group** | *P*6_3_/*mcm* | | |
| **Calculated unit cell** | *a* = *b* = 46.3989 Å, *c* = 9.4727 Å  *α* = *β* = 90°, *γ* = 120° | | |
| **Atom** | **x** | **y** | **z** |
| S1 | 0.25226 | 0.62613 | 0.5 |
| H2 | 0.26581 | 0.64083 | 0.48914 |
| N1 | 0.33333 | 0.66667 | 0.5 |
| N2 | 0.15926 | 0.74001 | 0.51228 |
| C1 | 0.31512 | 0.68488 | 0.5 |
| C2 | 0.28021 | 0.66894 | 0.48917 |
| C3 | 0.2635 | 0.68659 | 0.47879 |
| H3 | 0.23606 | 0.67319 | 0.45234 |
| C4 | 0.27953 | 0.72047 | 0.5 |
| C5 | 0.26112 | 0.73888 | 0.5 |
| C6 | 0.22687 | 0.72249 | 0.5294 |
| H6 | 0.21342 | 0.69519 | 0.55866 |
| C7 | 0.20959 | 0.73958 | 0.52268 |
| C8 | 0.22666 | 0.77334 | 0.5 |
| H23 | 0.21264 | 0.78737 | 0.5 |
| C9 | 0.17375 | 0.72311 | 0.5388 |
| H9 | 0.15858 | 0.69623 | 0.57354 |
| C10 | 0.12508 | 0.72934 | 0.52705 |
| C11 | 0.10085 | 0.6959 | 0.53744 |
| H11 | 0.10764 | 0.67601 | 0.51726 |
| C12 | 0.06861 | 0.68724 | 0.57243 |
| H12 | 0.04879 | 0.66014 | 0.58026 |
| C13 | 0.06056 | 0.71186 | 0.59799 |
| C14 | 0.08398 | 0.745 | 0.57554 |
| H14 | 0.07671 | 0.76476 | 0.58715 |
| C15 | 0.11613 | 0.75363 | 0.53904 |
| H15 | 0.13521 | 0.7806 | 0.51889 |
| C16 | 0.02972 | 0.70303 | 0.67395 |
| C17 | 0 | 0.68973 | 0.59727 |
| C18 | 0 | 0.68971 | 0.43474 |
| H18 | 0.02192 | 0.71201 | 0.39175 |
| H19 | 0 | 0.66737 | 0.39488 |
| O1 | 0.0291 | 0.5206 | 0.6211 |
| O2 | 0.0662 | 0.5802 | 0.4305 |
| O3 | 0.0805 | 0.5741 | 0.6389 |

**Table S8.** Fractional atomic coordinates for **T-COF** modelled based on the **zyg** net.

| **T-COF** | | | |
| --- | --- | --- | --- |
| **Space group** | *P*6_3_/*mcm* | | |
| **Calculated unit cell** | *a* = *b* = 50.5493 Å, *c* = 9.1344 Å  *α* = *β* = 90°, *γ* = 120° | | |
| **Atom** | **x** | **y** | **z** |
| N1 | 0.15244 | 0.74483 | 0.0083 |
| C1 | 0.30144 | 0.65072 | 0 |
| H1 | 0.2768 | 0.6384 | 0 |
| C2 | 0.31714 | 0.68286 | 0 |
| C3 | 0.30002 | 0.69998 | 0 |
| C4 | 0.31264 | 0.72916 | 0.06405 |
| H4 | 0.33462 | 0.73965 | 0.11769 |
| C5 | 0.29676 | 0.74518 | 0.06317 |
| H5 | 0.30714 | 0.76741 | 0.11481 |
| C6 | 0.2676 | 0.7324 | 0 |
| C7 | 0.2506 | 0.7494 | 0 |
| C8 | 0.21877 | 0.73389 | 0.01988 |
| H8 | 0.20651 | 0.70944 | 0.03779 |
| C9 | 0.20252 | 0.74967 | 0.01732 |
| C10 | 0.21845 | 0.78155 | 0 |
| H10 | 0.20608 | 0.79392 | 0 |
| C11 | 0.16903 | 0.73233 | 0.03491 |
| H11 | 0.15871 | 0.70901 | 0.07329 |
| C12 | 0.11981 | 0.73006 | 0.02437 |
| C13 | 0.10113 | 0.69805 | 0.03233 |
| H13 | 0.11027 | 0.683 | 0.01532 |
| C14 | 0.07027 | 0.68501 | 0.06829 |
| H14 | 0.05669 | 0.66053 | 0.08394 |
| C15 | 0.05756 | 0.70363 | 0.09517 |
| C16 | 0.0754 | 0.73526 | 0.07425 |
| H16 | 0.06589 | 0.74991 | 0.09546 |
| C17 | 0.10635 | 0.74834 | 0.03869 |
| H17 | 0.12028 | 0.77295 | 0.03064 |
| C18 | 0.02783 | 0.69056 | 0.17247 |
| C19 | 0 | 0.67656 | 0.09438 |
| C20 | 0 | 0.67684 | -0.07099 |
| H20 | 0.02019 | 0.67657 | -0.1164 |
| H21 | 0 | 0.69764 | -0.10981 |

**Table S9.** Fractional atomic coordinates for **T-COF** derived from Rietveld refinement.

| **T-COF** | | | |
| --- | --- | --- | --- |
| **Space group** | *P*6_3_/*mcm* | | |
| **Calculated unit cell** | *a* = *b* = 49.6999 Å, *c* = 9.1212 Å  *α* = *β* = 90°, *γ* = 120° | | |
| **Atom** | **x** | **y** | **z** |
| N1 | 0.15171 | 0.74817 | -0.00453 |
| C1 | 0.30094 | 0.65047 | 0 |
| H1 | 0.27589 | 0.63795 | 0 |
| C2 | 0.31694 | 0.68306 | 0 |
| C3 | 0.29965 | 0.70035 | 0 |
| C4 | 0.27015 | 0.68756 | -0.06541 |
| H4 | 0.25958 | 0.66528 | -0.11986 |
| C5 | 0.25393 | 0.70367 | -0.06475 |
| H5 | 0.23146 | 0.69318 | -0.11768 |
| C6 | 0.26686 | 0.73314 | 0 |
| C7 | 0.24967 | 0.75033 | 0 |
| C8 | 0.21711 | 0.7343 | 0.01278 |
| H8 | 0.20435 | 0.7093 | 0.02599 |
| C9 | 0.20079 | 0.75042 | 0.00911 |
| C10 | 0.21715 | 0.78285 | 0 |
| H10 | 0.20457 | 0.79543 | 0 |
| C11 | 0.16677 | 0.73349 | 0.01859 |
| H11 | 0.15498 | 0.70939 | 0.05212 |
| C12 | 0.11906 | 0.7359 | 0.01216 |
| C13 | 0.09795 | 0.70386 | 0.02365 |
| H13 | 0.10503 | 0.6868 | 0.00574 |
| C14 | 0.0674 | 0.69353 | 0.06549 |
| H14 | 0.05178 | 0.66894 | 0.0832 |
| C15 | 0.05769 | 0.71501 | 0.09499 |
| C16 | 0.07786 | 0.7467 | 0.07099 |
| H16 | 0.07048 | 0.76342 | 0.09379 |
| C17 | 0.10845 | 0.75705 | 0.02898 |
| H17 | 0.12449 | 0.78175 | 0.01874 |
| C18 | 0.02809 | 0.70437 | 0.17247 |
| C19 | 0 | 0.69091 | 0.09411 |
| C20 | 0 | 0.69193 | -0.0717 |
| H20 | 0.02049 | 0.69189 | -0.11912 |
| H21 | 0 | 0.71326 | -0.10764 |

**Table S10.** Fractional atomic coordinates for **N-COF** modelled based on the **rht** net.

| **N-COF** | | | |
| --- | --- | --- | --- |
| **Space group** | *F*432 | | |
| **Calculated unit cell** | *a* = *b* = *c* = 69.6878 Å  *α* = *β* = *γ* = 90° | | |
| **Atom** | **x** | **y** | **z** |
| N1 | 0.67982 | 0.82018 | 0.32018 |
| N2 | 0.58949 | 0.71185 | 0.35801 |
| N3 | 0.58938 | 0.64126 | 0.28852 |
| C1 | 0.67136 | 0.80358 | 0.32927 |
| C2 | 0.66441 | 0.78827 | 0.31815 |
| H2 | 0.66577 | 0.78858 | 0.30268 |
| C3 | 0.65569 | 0.77247 | 0.32692 |
| H3 | 0.65057 | 0.76081 | 0.31801 |
| C4 | 0.65375 | 0.77167 | 0.34699 |
| C5 | 0.66085 | 0.78695 | 0.35808 |
| H5 | 0.65929 | 0.78691 | 0.37354 |
| C6 | 0.66954 | 0.80273 | 0.34932 |
| H6 | 0.67455 | 0.81448 | 0.35816 |
| C7 | 0.64408 | 0.75515 | 0.35635 |
| C8 | 0.62724 | 0.74734 | 0.34848 |
| H8 | 0.62133 | 0.75336 | 0.33542 |
| C9 | 0.61759 | 0.73225 | 0.35768 |
| C10 | 0.625 | 0.72481 | 0.37491 |
| H10 | 0.61759 | 0.7132 | 0.38212 |
| C11 | 0.64191 | 0.73237 | 0.38288 |
| C12 | 0.65135 | 0.74747 | 0.37351 |
| H12 | 0.66445 | 0.75324 | 0.37959 |
| C13 | 0.59962 | 0.72482 | 0.34921 |
| H13 | 0.59512 | 0.73059 | 0.33555 |
| C14 | 0.57153 | 0.70382 | 0.35171 |
| C15 | 0.56114 | 0.71113 | 0.33594 |
| H15 | 0.56618 | 0.72322 | 0.32768 |
| C16 | 0.54363 | 0.70292 | 0.33069 |
| H16 | 0.53583 | 0.70865 | 0.31852 |
| C17 | 0.53613 | 0.68746 | 0.34111 |
| C18 | 0.54633 | 0.68019 | 0.35683 |
| H18 | 0.54068 | 0.66822 | 0.36496 |
| C19 | 0.56389 | 0.68836 | 0.36207 |
| H19 | 0.5716 | 0.68264 | 0.37429 |
| C20 | 0.5358 | 0.65817 | 0.31229 |
| C21 | 0.54786 | 0.64539 | 0.32206 |
| H21 | 0.54367 | 0.63969 | 0.3359 |
| C22 | 0.56543 | 0.63999 | 0.31402 |
| C23 | 0.57125 | 0.64738 | 0.29625 |
| C24 | 0.55892 | 0.66001 | 0.28634 |
| H24 | 0.56252 | 0.66554 | 0.27231 |
| C25 | 0.54138 | 0.6654 | 0.29439 |
| C26 | 0.51741 | 0.66443 | 0.32092 |
| C27 | 0.51762 | 0.6788 | 0.33523 |
| C28 | 0.50025 | 0.68588 | 0.34292 |
| C29 | 0.50095 | 0.70094 | 0.35862 |
| H29a | 0.50961 | 0.69552 | 0.37084 |
| H29b | 0.48667 | 0.70476 | 0.36446 |
| H29c | 0.50767 | 0.71425 | 0.35299 |
| C30 | 0.59888 | 0.64985 | 0.27495 |
| H30 | 0.59371 | 0.66314 | 0.26883 |
| H22 | 0.57462 | 0.6301 | 0.32171 |
| H25 | 0.53212 | 0.67523 | 0.28668 |

**Table S11.** Fractional atomic coordinates for **N-COF** modelled based on the **stp** net.

| **N-COF** | | | |
| --- | --- | --- | --- |
| **Space group** | *P*6 | | |
| **Calculated unit cell** | *a* = *b* = 52.2987 Å, *c* = 13.8831 Å  *α* = *β* = 90°, *γ* = 120° | | |
| **Atom** | **x** | **y** | **z** |
| N1 | 0.5885 | 0.4703 | 1.316 |
| H1 | 0.5937 | 0.4821 | 1.3646 |
| N2 | 0.5878 | 0.4695 | 0.6846 |
| H2 | 0.5936 | 0.4816 | 0.6369 |
| C1 | 0.5747 | 0.4763 | 1.2368 |
| C2 | 0.5519 | 0.4533 | 1.1837 |
| H2A | 0.5459 | 0.4306 | 1.1964 |
| C3 | 0.5349 | 0.4593 | 1.1198 |
| H3 | 0.5161 | 0.4411 | 1.0848 |
| C4 | 0.5408 | 0.4883 | 1.1064 |
| C5 | 0.5644 | 0.5114 | 1.1563 |
| H5 | 0.5689 | 0.5339 | 1.148 |
| C6 | 0.5809 | 0.5054 | 1.2219 |
| H6 | 0.598 | 0.5232 | 1.2636 |
| C7 | 0.5199 | 0.4941 | 1.0514 |
| C8 | 0.5198 | 0.494 | 0.9497 |
| H11A | 0.492 | 0.4801 | 1.2331 |
| H11B | 0.4881 | 0.508 | 1.2331 |
| H11C | 0.5199 | 0.5119 | 1.2331 |
| H12A | 0.5151 | 0.4961 | 0.768 |
| H12B | 0.5039 | 0.5189 | 0.768 |
| H12C | 0.4811 | 0.4849 | 0.768 |
| C13 | 0.5405 | 0.4879 | 0.8946 |
| C14 | 0.5338 | 0.4587 | 0.8794 |
| H14 | 0.5147 | 0.4406 | 0.9133 |
| C15 | 0.5506 | 0.4525 | 0.8153 |
| H15 | 0.5441 | 0.4297 | 0.8013 |
| C16 | 0.574 | 0.4755 | 0.7638 |
| C17 | 0.5809 | 0.5047 | 0.7804 |
| H17 | 0.5984 | 0.5225 | 0.7399 |
| C18 | 0.5645 | 0.5109 | 0.8462 |
| H18 | 0.5695 | 0.5335 | 0.8558 |
| C19 | 0.5922 | 0.4472 | 0.6793 |
| H19 | 0.5878 | 0.4328 | 0.7406 |
| C20 | 0.6019 | 0.4403 | 0.5878 |
| C21 | 0.5973 | 0.4508 | 0.5 |
| H21 | 0.5877 | 0.4649 | 0.5001 |
| C22 | 0.6131 | 0.421 | 0.5872 |
| H22 | 0.6167 | 0.413 | 0.6549 |
| C23 | 0.6199 | 0.4121 | 0.4998 |
| C24 | 0.6319 | 0.3917 | 0.4995 |
| C25 | 0.6199 | 0.3669 | 0.5601 |
| H25 | 0.6015 | 0.3621 | 0.6071 |
| C26 | 0.6312 | 0.3477 | 0.5595 |
| H26 | 0.6217 | 0.3287 | 0.607 |
| C27 | 0.6549 | 0.3531 | 0.4989 |
| C28 | 0.6669 | 0.3779 | 0.4384 |
| H28 | 0.6851 | 0.3824 | 0.3908 |
| C29 | 0.6555 | 0.397 | 0.4384 |
| H29 | 0.6653 | 0.4161 | 0.3916 |
| C30 | 0.5937 | 0.4485 | 1.3203 |
| H30 | 0.59 | 0.4346 | 1.258 |
| C31 | 0.6028 | 0.4412 | 1.412 |
| C32 | 0.6143 | 0.422 | 1.4124 |
| H32 | 0.6182 | 0.4143 | 1.3446 |
| N3 | 0.66667 | 0.33333 | 0.4987 |
| C9 | 0.5 | 0.5 | 0.8993 |
| C10 | 0.5 | 0.5 | 1.1018 |
| C11 | 0.5 | 0.5 | 1.2101 |
| C12 | 0.5 | 0.5 | 0.791 |

**References**

1. Qian C, Xu S-Q, Jiang G-F *et al.* Precision construction of 2D heteropore covalent organic frameworks by a multiple-linking-site strategy. *Chem. Eur. J.* 2016; **22**: 17784-17789.
2. Coelho Software, Brisbane, Australia. 2012.
3. Frisch, M. J. et al. Gaussian 16, Revision C.01, Gaussian Inc., Wallingford CT. 2019.
4. Grimme S, Antony J, Ehrlich S *et al.* A consistent and accurate ab initio parametrization of density functional dispersion correction (DFT-D) for the 94 elements H-Pu. *J. Chem. Phys.* 2010; **132**: 154104.
5. Mammen M, Shakhnovich E I, Deuth J M *et al.* Estimating the entropic cost of self-assembly of multiparticle hydrogen-bonded aggregates based on the cyanuric acid·melamine lattice. *J. Org. Chem.* 1998; **63**: 3821-3830.
6. Marenich A V, Cramer C J, Truhlar D G. Universal solvation model based on solute electron density and on a continuum model of the solvent defined by the bulk dielectric constant and atomic surface tensions. *J. Phys. Chem. B*. 2009; **113**: 6378-6396.
7. Peintinger M F, Oliveira D V, Bredow T. Consistent gaussian basis sets of triple‐zeta valence with polarization quality for solid‐state calculations. *J. Comput. Chem.* 2012; **34**: 451-459.
8. Tan F, Zheng Y, Zhou Z *et al.* Aqueous synthesis of covalent organic frameworks as photocatalysts for hydrogen peroxide production. *CCS Chem.* 2022; **4**: 3751-3761.
9. Kou M, Wang Y, Xu Y *et al.* Molecularly engineered covalent organic frameworks for hydrogen peroxide photosynthesis. *Angew. Chem. Int. Ed.* 2022; **61**: e202200413.
10. Zhao W, Yan P, Li B *et al.* Accelerated synthesis and discovery of covalent organic framework photocatalysts for hydrogen peroxide production. *J. Am. Chem. Soc.* 2022; **144**: 9902-9909.
11. Wu C, Teng Z, Yang C *et al.* Polarization engineering of covalent triazine frameworks for highly efficient photosynthesis of hydrogen peroxide from molecular oxygen and water. *Adv. Mater.* 2022; **34**: 2110266.
12. Deng M, Sun J, Laemont A *et al.* Extending the π-conjugation system of covalent organic frameworks for more efficient photocatalytic H_2_O_2_ production. *Green Chem.* 2023; **25**: 3069-3076.
13. Das P, Chakraborty G, Roeser J *et al.* Integrating Bifunctionality and Chemical Stability in Covalent Organic Frameworks via One-Pot Multicomponent Reactions for Solar-Driven H_2_O_2_ Production. *J. Am. Chem. Soc.* 2023; **145**: 2975-2984.
14. Das P, Roeser J, Thomas A. Solar light driven H_2_O_2_ production and selective oxidations using a covalent organic framework photocatalyst prepared by a multicomponent reaction. *Angew. Chem. Int. Ed.* 2023; **62**: e202304349.
15. Liu Y, Han W-K, Chi W *et al.* Substoichiometric covalent organic frameworks with uncondensed aldehyde for highly efficient hydrogen peroxide photosynthesis in pure water. *Appl. Catal. B Environ.* 2023; **331**: 122691.
16. Cheng H, Lv H, Cheng J *et al.* Rational design of covalent heptazine frameworks with spatially separated redox centers for high‐efficiency photocatalytic hydrogen peroxide production. *Adv. Mater.* 2021; **34**: 2107480.
17. Chen D, Chen W, Wu Y *et al.* Covalent organic frameworks containing dual O_2_ reduction centers for overall photosynthetic hydrogen peroxide production. *Angew. Chem. Int. Ed.* 2023; **135**: e202217479.
18. Liao Q, Sun Q, Xu H *et al.* Regulating relative nitrogen locations of diazine functionalized covalent organic frameworks for overall H_2_O_2_ photosynthesis. *Angew. Chem. Int. Ed.* 2023; **62**: e202310556.
19. Chai S, Chen X, Zhang X *et al.* Rational design of covalent organic frameworks for efficient photocatalytic hydrogen peroxide production. *Environ. Sci.: Nano*. 2022; **9**: 2464-2469.
20. Li L, Xu L, Hu Z *et al.* Enhanced mass transfer of oxygen through a gas–liquid–solid interface for photocatalytic hydrogen peroxide production. *Adv. Funct. Mater.* 2021; **31**: 2106120.
21. Chang J N, Li Q, Shi J W *et al.* Oxidation‐reduction molecular junction covalent organic frameworks for full reaction photosynthesis of H_2_O_2_. *Angew. Chem. Int. Ed.* 2023; **62**: e202218868.
22. Qin C, Wu X, Tang L *et al.* Dual donor-acceptor covalent organic frameworks for hydrogen peroxide photosynthesis. *Nat. Commun.* 2023; **14**: 5238.
